# Ets2 in Tumor Fibroblasts Promotes Angiogenesis in Breast Cancer

**DOI:** 10.1371/journal.pone.0071533

**Published:** 2013-08-16

**Authors:** Julie A. Wallace, Fu Li, Subhasree Balakrishnan, Carmen Z. Cantemir-Stone, Thierry Pecot, Chelsea Martin, Raleigh D. Kladney, Sudarshana M. Sharma, Anthony J. Trimboli, Soledad A. Fernandez, Lianbo Yu, Thomas J. Rosol, Paul C. Stromberg, Robert Lesurf, Michael Hallett, Morag Park, Gustavo Leone, Michael C. Ostrowski

**Affiliations:** 1 Department of Molecular and Cellular Biochemistry, College of Medicine, The Ohio State University, Columbus, Ohio, United States of America; 2 Department of Molecular Genetics, College of Biological Sciences, The Ohio State University, Columbus, Ohio, United States of America; 3 Department of Molecular Virology, Immunology and Medical Genetics, The Ohio State University, Columbus, Ohio, United States of America; 4 The Ohio State University Computer Science and Engineering, The Ohio State University Biomedical Informatics, The Ohio State University, Columbus, Ohio, United States of America; 5 Center for Biostatistics, The Ohio State University, Columbus, Ohio, United States of America; 6 Department of Veterinary Biosciences, College of Veterinary Medicine, The Ohio State University, Columbus, Ohio, United States of America; 7 Department of Biochemistry, Rosalind and Morris Goodman Cancer Centre, McGill University, Québec, Canada; 8 McGill Centre for Bioinformatics, McGill University, Québec, Canada; 9 Department of Oncology, McGill University, Québec, Canada; 10 Tumor Microenvironment Program, Comprehensive Cancer Center, The Ohio State University, Columbus, Ohio, United States of America; University of Alabama at Birmingham, United States of America

## Abstract

Tumor fibroblasts are active partners in tumor progression, but the genes and pathways that mediate this collaboration are ill-defined. Previous work demonstrates that *Ets2* function in stromal cells significantly contributes to breast tumor progression. Conditional mouse models were used to study the function of *Ets2* in both mammary stromal fibroblasts and epithelial cells. Conditional inactivation of *Ets2* in stromal fibroblasts in *PyMT* and *ErbB2* driven tumors significantly reduced tumor growth, however deletion of *Ets2* in epithelial cells in the *PyMT* model had no significant effect. Analysis of gene expression in fibroblasts revealed a tumor- and *Ets2-*dependent gene signature that was enriched in genes important for ECM remodeling, cell migration, and angiogenesis in both *PyMT* and *ErbB2* driven-tumors. Consistent with these results, *PyMT* and *ErbB2* tumors lacking *Ets2* in fibroblasts had fewer functional blood vessels, and *Ets2* in fibroblasts elicited changes in gene expression in tumor endothelial cells consistent with this phenotype. An *in vivo* angiogenesis assay revealed the ability of *Ets2* in fibroblasts to promote blood vessel formation in the absence of tumor cells. Importantly, the *Ets2*-dependent gene expression signatures from both mouse models were able to distinguish human breast tumor stroma from normal stroma, and correlated with patient outcomes in two whole tumor breast cancer data sets. The data reveals a key function for *Ets2* in tumor fibroblasts in signaling to endothelial cells to promote tumor angiogenesis. The results highlight the collaborative networks that orchestrate communication between stromal cells and tumor cells, and suggest that targeting tumor fibroblasts may be an effective strategy for developing novel anti-angiogenic therapies.

## Introduction

Although the microenvironment of normal tissue can inhibit tumor growth, changes induced by tumor cells actually influence this microenvironment to promote tumor progression and metastasis [1], [2], [3]. For example, disruption of *Tgfbr2* in fibroblasts leads to growth and increased oncogenic potential of adjacent epithelial cells in stomach and prostate [4]. Cancer associated fibroblasts (CAFs) were also shown to mediate immune cell recruitment in an NF-κB dependent manner in a mouse model of squamous cell carcinoma [5]. In addition, recent evidence demonstrates that a human stromal gene signature can be correlated with tumor prognosis and evolution of breast cancer [6]. Stromal fibroblasts are one of the major components within the tumor microenvironment that drive a number of characteristics associated with tumor malignancy, including remodeling of the extracellular matrix, immune cell recruitment and modulation, angiogenesis and tumor cell proliferation and invasion [7], [8]. Identification of specific molecules and signaling pathways within this stroma which promote tumor initiation and progression is essential for the potential development of novel therapeutics to be used independently or in combination with other tumor cell-specific treatments [9].

*Ets2* (v-ets erythroblastosis virus E26 oncogene homologue 2) is an evolutionarily conserved proto-oncogene known to be overexpressed in a variety of human cancers including breast and prostate [10], [11]. As a downstream effector of the Ras/Raf/MAPK pathway, *Ets2* regulates the expression of a number of genes with potentially important functions in the tumor microenvironment, including growth factors, adhesion molecules, extracellular proteases and anti-apoptotic genes. In mouse mammary tumor models, *Ets2* was shown to support tumor growth from an undefined cell population in the tumor stroma [12], [13].

Previous work from our group has shown *Ets2* functions to promote tumor progression and metastasis in at least two compartments of the mammary tumor microenvironment, including tumor macrophages and breast stromal fibroblasts in which *Pten* was deleted [14], [15]. However, questions concerning both the fibroblast-specific and oncogene-dependent functions of *Ets2*, as well as the molecular and cellular mechanisms of action remain unanswered. To address these issues, we used a *Cre/loxP* genetic approach to investigate the role of *Ets2* in mammary epithelial cells and mammary fibroblasts in the context of *PyMT* and *ErbB2* driven tumors. This approach revealed that fibroblast *Ets2* promotes tumor growth and progression, whereas its function in epithelial cells is dispensable for tumor progression. Transcriptome profiling of both normal and tumor fibroblasts revealed tumor specific targets of *Ets2* including genes involved in matrix remodeling and angiogenesis in the context of both *PyMT* and *ErbB2*. Consistent with the gene expression results, loss of *Ets2* in tumor fibroblasts decreased tumor associated blood vessels in both models. Additionally, *Ets2* deficiency in tumor fibroblasts significantly impaired angiogenesis *in vivo* in matrigel plug assays. The fibroblast *Ets2* transcriptomes were represented in human breast cancer stroma and correlated with patient outcome in independent whole-tumor breast cancer gene expression data sets. These results indicate that *Ets2* functions in a tumor-dependent but tumor cell non-autonomous manner to affect tumor growth and angiogenesis.

## Methods

### Ethics Statement

The use of animals was in compliance with federal and University Laboratory Animal Resources at The Ohio State University regulations and was conducted under under protocol number 2007A0120-R1 which was reviewed and approved by the Ohio State University Institutional Animal Care and Use Committee.

### Tumor Studies

All mice used in these studies were inbred 10 generations to the FVB/N (F10) background. The generation of *Fsp-Cre* and *Ets2^loxP^* mice has been previously described [15], [16]. *Ets2^db^* mice were a gift from Dr. Robert Oshima (The Burnham Institute, La Jolla, California). *PyMT* tumor bearing mice were sacrificed 4 weeks after palpable tumors were identified. Excised tumors were weighed and processed for histological analysis. X-gal staining was done as described elsewhere [16]. *ErbB2* mice were sacrificed at 16 weeks of age, and all mammary glands were harvested and processed for histological analysis. The total area of tumor lesions was calculated using Zeiss AxioVision 3.1 software.

### Lectin Injections and Matrigel Plug Assay

Fluorescein or DyLight 594 Lycopersicon esculentum lectin (Vector Laboratories) was used to visualize blood vessels as described [17]. For the *in vivo* matrigel angiogenesis assays, 250,000 primary fibroblasts were mixed with ice cold growth factor reduced matrigel (BD Bioscience) and subcutaneously injected into the flanks of two month old FVB/N mice. Matrigel plugs were retrieved 5 days post injection for subsequent analysis.

### X-gal Staining, Immunostaining, Gelatinase *in situ* Zymography, and Microscopy Imaging and Quantification

X-gal staining was performed as previously described [15]. The following antibodies were used in this study: CD31 (BD Bioscience), Ki67 (Dako) and cleaved caspase- 3 (Cell Signaling). Appropriate secondary antibodies were used for IF or IHC as described elsewhere [15]. DQ-gelatin (Molecular Probes) was used for gelatinase *in situ* zymography as described by Mook *et al*. [18]. For CD31 and lectin quantification, images were made from multiple tumors in at least three different mice; a minimum of five images were taken per mouse from random tumor fields. Microscopic images for CD31, lectin and zymography quantification were acquired with a Zeiss Axiocam HRc camera and quantified using ImageJ (http://rsbweb.nih.gov/ij/), Fiji [19] or MetaMorph softwares, whereby the percentage of total positive area was calculated. For quantification of Ki67 and cleaved caspase 3 staining, images from random tumor fields were taken, and the total number of positive cells was represented as a percentage of the total number of tumor cells.

#### Whole mount staining

Inguinal mammary glands were isolated from 6 week or 14 days pregnant female mice and mounted on glass slides using a Q-tip to spread the tissue. Tissue was fixed using Carnoy’s fixative (60% ethanol, 30% chloroform and 10% acetic acid) at 4°C overnight, and then stained with carmine alum stain (Sigma) for 12 hours. After thorough rinsing in water, mammary glands were dehydrated with alcohol and preserved in xylene.

### Primary Mammary Fibroblast Culture, Endothelial Cell Isolation and Microarray Analysis

Mammary fibroblasts were isolated from female mice using gravity separation [20]. Briefly, mammary tissue was digested with collagenase/hyaluronidase overnight in a CO_2_ incubator. After neutralization, cells were separated by gravity five times. Supernatant containing stromal cells was plated with 10% FBS DMEM. Second passage cells were used for subsequent analysis. For endothelial cell isolation, mammary glands were digested with collagenase until a single cell suspension was achieved. Cells were then stained with fluorescently conjugated CD31 antibody (BD Pharmingen) and subjected to cell sorting. RNA was extracted with Trizol (Invitrogen) and purified with RNAeasy kit (Qiagen) following manufacturers’ instructions. Microarray profiling was performed with Affymetrix Mouse Genome 430A 2.0 or 430 2.0 GeneChips at the OSUCCC microarray facility. All microarray data has been deposited and can be accessed through the following links:

http://www.ncbi.nlm.nih.gov/geo/query/acc.cgi?token=dberdqwuosoegdi&acc=GSE16989

http://www.ncbi.nlm.nih.gov/geo/query/acc.cgi?token=flmxjguyowqmojq&acc=GSE44166

http://www.ncbi.nlm.nih.gov/geo/query/acc.cgi?token=txmnnecmegeaopw&acc=GSE44118

Differentially expressed genes were identified by the WEDGE^++^ method [21] or Robust Multichip Average method (RMA) [22]. Quantitative RT procedures are described elsewhere [23]. Primers and probes used for real time PCR are available in Table S6.

### Western Blot

Western blotting was performed as previously described [15].

#### Gene Set Enrichment Analysis (GSEA)

GSEA v2.0 was used to determine the enrichment of functional categories in our gene expression data [24]. Gene sets were obtained from indicated categories in ToppGene Suite [25]. Statistical significance was determined using 1,000 random permutations of each gene set to obtain a nominal P value.

#### Generating the human stroma heat map using the *Ets2* gene lists

Of the 107 and 69 genes regulated by *Ets2* in *PyMT* and *ErbB2* derived tumor fibroblasts, 88 and 61 had human orthologs identified using Ensembl and MGI databases. When queried against the McGill Cancer Center’s Breast Stroma Microarray data (GSE9014 and GSE4823), 85 and 57 genes were also represented on the Agilent custom array used by the McGill group. To identify the genes most differentially expressed in human tumor stroma versus normal stroma, a variance cutoff of>0.5 was applied across the multiple human samples. The 38 *PyMT* derived and 36 *ErbB2* derived genes which met this cutoff criterion were subsequently used to generate a heat map for the human stroma dataset (52 normal stroma and 49 tumor stroma samples). The separation of normal stroma and tumor stroma was significant using gene signatures from both models (P = 0.00085 for *PyMT* and 0.00004 for *ErbB2*), as determined by 10,000 random permutations using the one sided Wilcoxon rank sum test. Similar analysis was used for our *PyMT* derived *Ets2* dependent endothelial cell data, with 22 of 33 human orthologs represented in the McGill breast stroma data. In this case, the separation of normal stroma and tumor stroma was not found to be significant (p-value = 0.1014).

#### Kaplan-meier survival analysis

The *Ets2* tumor fibroblast 38 and 36 gene signatures as well as the endothelial 22 gene signature were used to query gene expression data from three independent breast cancer microarrays - Rosetta (NKI) dataset, Stockholm (GSE1456) and the McGill University (GSE9014) dataset. A Cox proportional hazards model associated to a L2 norm constraint [26] was applied to estimate the coefficients β associated to each gene from the human orthologs corresponding to the *Ets2*- tumor fibroblast 38 or 36 gene signatures. A prognostic index for each patient is defined as the sum of the gene expression levels pondered by the estimated coefficients β. A 10-fold cross validation was used to predict the outcome for each patient: the patients are randomly divided into 10 groups; 9 groups are chosen to form the training set while the last group represents the test set. The coefficients β are estimated from the training set and the prognostic index for each patient in the test set is computed. This step is repeated 10 times to consider each group of patients as the test set once. The 10-fold cross validation is performed 10 times in order to be independent of the random partitioning. A prognostic index threshold is then defined as the 50 percentile of all prognostic indexes. All the patients that have a prognostic index superior (respectively inferior) to this threshold are considered as high risk (respectively low risk) patients. A log-rank test is finally applied to evaluate the statistical significance between the two groups.

### Statistical Analysis

**Figure 1:**
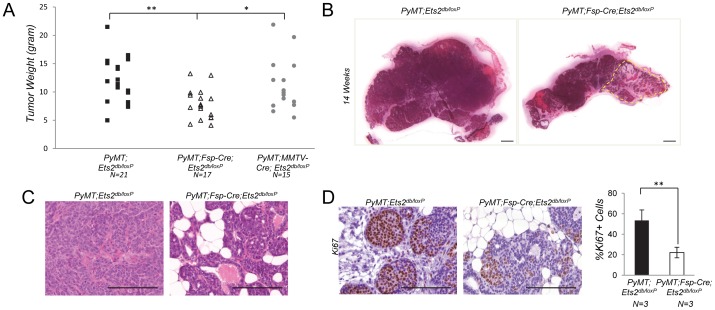
*Ets2* ablation in stromal fibroblasts restricts *PyMT* driven mammary tumorigenesis. **A.** Dissected tumor weight in grams isolated from 14 week old *PyMT;Ets2^db/loxP^* (n = 21, 12.36±3.8), *PyMT;Fsp-Cre;Ets2^db/loxP^* (n = 17, 7.83±2.7), and *PyMT;MMTV-Cre;Ets2^db/loxP^* (n = 15, 11.3±4.7) mice (**P<0.01, *P<0.05, adjusted P-values were obtained from an ANOVA model assuming unequal variance with Holm’s methods). **B.** Representative H&E stained mammary glands from 14 week old *PyMT;Ets2^db/loxP^* and *PyMT;Fsp-Cre;Ets2^db/loxP^* mice. Area outlined in yellow represents less advanced tumor progression. Scale bar, 2mm. **C.** Representative histological sections from mammary glands of 10 week old *PyMT;Ets2^db/loxP^* and *PyMT;Fsp-Cre;Ets2^db/loxP^* mice. Scale bar, 50 µm. **D.** Left: representative IHC staining for Ki67 in mammary glands of 10 week old *PyMT;Ets2^db/loxP^* and *PyMT;Fsp-Cre;Ets2^db/loxP^* mice. Scale bar, 50 µm. Right: graph represents percentage of Ki67 positive epithelial cells (n = 3, bars represent means ± SD, **P<0.01, unpaired Welch’s t-test assuming unequal variance).

**Figure 2:**
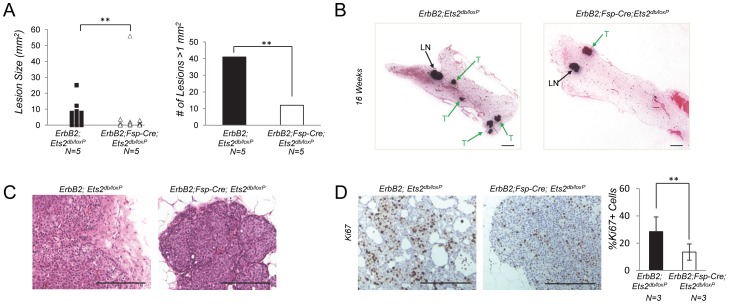
*Ets2* deletion in stromal fibroblasts reduces size and number of tumor lesions in *ErbB2* breast cancer model. **A.** Left: graph represents carcinoma lesion sizes in mm^2^ from 16 week old *ErbB2;Ets2^db/loxP^* (n = 5, 2.28±4.16) and *ErbB2;Fsp-Cre;Ets2^db/loxP^* (n = 5, 1.83±7.96) mice (**P<0.01, Non-parametric Mann-Whitney test). Right: graph represents number of lesions larger than one mm^2^ in mammary glands of 16 week old *ErbB2;Ets2^db/loxP^* (n = 5) and *ErbB2;Fsp-Cre;Ets2^db/loxP^* (n = 5) mice (**P<0.01, Chi Square test). **B.** Representative H&E stained mammary glands from 16 week old *ErbB2;Ets2^db/loxP^* and *ErbB2;Fsp-Cre;Ets2^db/loxP^* mice. Scale bar, 2mm. LN, lymph node. T, tumor. **C.** Representative histological sections from mammary glands of 16 week old *ErbB2;Ets2^db/loxP^* and *ErbB2;Fsp-Cre;Ets2^db/loxP^* mice. Scale bar, 50 µm. **D.** Left: representative IHC staining for Ki67 in mammary glands from 16 week old *ErbB2;Ets2^db/loxP^* and *ErbB2;Fsp-Cre;Ets2^db/loxP^* mice. Scale bar, 50 µm. Right: graph represents percentage of Ki67 positive epithelial cells (n = 3, bars represent means ± SD, **P<0.01, Welch’s t-test assuming unequal variance).

Number of animals and experiments are as indicated in the figures. Analysis of variance (ANOVA) model assuming unequal variance with Holm’s methods was used to study the differences among groups in the *PyMT* tumor study (Fig. 1A). A non-parametric Mann-Whitney test was used to compare lesion sizes in *ErbB2* tumors (Fig. 2A, left panel). A Chi square test was used to compare the number of *ErbB2* lesions larger than 1mm^2^ (Fig. 2A, right panel). A Welch’s t-test assuming unequal variance was used to calculate p-values for Ki67 staining and all other immunofluorescence staining.

## Results and Discussion

### Ets2 Deletion Specifically Effects Tumor Progression from the Fibroblast Compartment in Multiple Breast Cancer Models

The ablation of a conditional *Ets2^loxP^* allele [27] was studied both in mammary stromal fibroblasts and in mammary tumor epithelial cells in the *MMTV-PyMT* (*PyMT*) mammary cancer model [28]. For this purpose, Fibroblast Specific Protein 1 promoter-Cre (*Fsp-Cre*) was used for specific deletion in tumor fibroblasts, while *MMTV-Cre* was used to target epithelial cells [15], [29]. Initial experiments indicated that deletion of both alleles in homozygous *Ets2^loxP/loxP^* mice was inefficient with several different Cre-driver lines [14], [15], including *Fsp-Cre* (data not shown). In order to facilitate the complete ablation of *Ets2* within the fibroblast and epithelial compartments, mice were generated carrying the *Cre* transgene along with one conditional *Ets2^loxP^* allele and a conventional *Ets2* knockout allele targeting the DNA-binding domain, *Ets2^db^* [30]. We evaluated tumor development in both *PyMT;Fsp-Cre;Ets2^db/loxP^* and *PyMT;MMTV-Cre;Ets2^db/loxP^* experimental groups and in control *PyMT;Ets2^db/loxP^* mice. No difference was observed in tumor initiation; however tumor progression was significantly impeded by the ablation of *Ets2* specifically in mammary fibroblasts. The average weight of tumors 30 days after tumor initiation in *PyMT;Fsp-Cre;Ets2^db/loxP^* mice was 1.5-fold lower than in either control *PyMT;Ets2^db/loxP^* or *PyMT;MMTV-Cre;Ets2^db/loxP^* mice (P<0.01, Fig. 1A). Additionally, H&E staining of inguinal mammary glands from control *PyMT;Ets2^db/loxP^* mice showed solid tumors throughout the mammary gland, whereas tumors had not invaded the entire fat pad of *PyMT;Fsp-Cre;Ets2^db/loxP^* mammary glands (Fig. 1B, yellow outline indicates area with less tumor invasion).

The specificities of both *Fsp-Cre* and *MMTV-Cre* were confirmed by introducing the *Rosa26^LoxP^* reporter gene into a subset of the experimental mice [31]. X-gal staining of mammary tumor sections confirmed the stromal- or epithelial-specific expression of the respective *Cre* transgenes (Fig. S1A). PCR-based analysis of genomic DNA from epithelial cells isolated from *PyMT;Ets2^db/loxP^* or *PyMT;MMTV-Cre;Ets2^db/loxP^* mice demonstrated efficient recombination of the single *Ets2^loxP^* allele (Fig. S1B). Western blot assays confirmed the loss of ETS2 [32] protein in mammary stromal fibroblasts isolated from *PyMT;Fsp-Cre;Ets2^db/loxP^* mice (Fig. S1C). Cultured mammary fibroblasts stained with anti-phospho-Ets2^pT72^ antibody further demonstrated the loss of ETS2 activation in fibroblasts from *PyMT;Fsp-Cre;Ets2^db/loxP^* mice [15] (Fig. S1D). Interestingly, whole mount analysis of normal developing and pregnant mammary glands showed similar branching of epithelial ducts in both *Ets2^db/loxP^* and *Fsp-Cre;Ets2^db/loxP^* animals (Fig. S1F, S1G), suggesting *Ets2* expression in fibroblasts was not essential for proper mammary gland development. Additionally, whole mount analysis of mammary glands isolated form 6-week old *PyMT;Ets2^db/loxP^* animals revealed extensive neoplastic progression, however tumors in mammary glands from *PyMT;Fsp-Cre;Ets2^db/loxP^* mice remained restricted to the nipple area, with little or no invasion into and beyond the mammary lymph node (Fig. S1H).

Careful examination of tumor histology revealed complete disruption of the basement membrane and invasive carcinoma in tumors from *PyMT;Ets2^db/loxP^* mice, while many tumor lesions in *PyMT;Fsp-Cre;Ets2^db/loxP^* mammary glands were restricted to the confines of the basement membrane (Fig. 1C). As assessed by Ki67 staining, there was an approximate 2.5 fold reduction in epithelial cell proliferation in tumors with *Ets2* deleted in the fibroblast compartment (P<0.01, Fig. 1D). Staining with cleavedcaspase- 3 failed to detect any difference in apoptosis between genetic groups (Fig. S1E), indicating that stromal *Ets2* impacts tumor growth through modulation of epithelial proliferation.

To determine if fibroblast *Ets2* function was oncogene dependent, we combined the *MMTV-ErbB2* oncogene with *Ets2^db/loxP^* and *Fsp-Cre;Ets2^db/loxP^* mice. Since an expected delay in tumor progression was predicted based on our results in the *PyMT* model, mice harboring the *ErbB2* oncogene were harvested at 16 weeks of age, just before palpable tumors were detected in this model. Using consecutive histological sections, we calculated the number and sizes of carcinoma lesions. The *ErbB2;Fsp-Cre;Ets2^db/loxP^* mice had nearly half as many overall lesions compared to those found in *ErbB2;Ets2^db/loxP^* mice (P<0.01, Fig. 2A, left panel, Fig. 2B). The lesions were also significantly smaller in *ErbB2;Fsp-Cre;Ets2^db/loxP^* compared to controls (Fig. 2A). Closer examination of the data revealed a three-fold decrease in the number of lesions>1mm^2^ in size in mammary glands from *ErbB2;Fsp-Cre;Ets2^db/loxP^* mice (P<0.01, Fig. 2A, right panel). As illustrated by higher magnification H&E staining, deletion of *Ets2* in tumor fibroblasts significantly reduced the expansion of carcinoma lesions into the surrounding fat pad tissue (Fig. 2C). As in the *PyMT* model, Ki67 staining revealed a 2-fold decrease in proliferation of epithelial cells surrounded by *Ets2* null fibroblasts, implicating a similar mechanism of controlling tumor growth in this model as well (Fig. 2D). Collectively, these data support a stromal fibroblast-specific function for *Ets2* in facilitating the progression and invasion of mammary tumors induced by both *PyMT* and *ErbB2* epithelial expression.

### Gene Expression Changes Reveal the Tumor Specific Effects of *Ets2*

To uncover the molecular mechanisms underlying *Ets2* function in stromal fibroblasts in both *PyMT* and *ErbB2* induced tumors, we profiled RNA isolated from primary mammary fibroblasts for changes in global gene expression (Affymetrix Mouse Genome 430A 2.0 GeneChip platform or 430 2.0 GeneChip platform). Fibroblasts were isolated when tumors were not yet invasive, from 9-week old mice in the *PyMT* model and from 16-week old mice in the *ErbB2* model. Fibroblasts from *Ets2^db/loxP^* and *Fsp-Cre;Ets2^db/loxP^* control mice were also isolated at the same time points and RNA was subjected to gene expression profiling. Assessment of cultured fibroblast purity has been previously described [15]; staining of cultured mammary fibroblasts with both vimentin and anti-phospho-Ets2^pT72^ antibodies confirmed both the enrichment for fibroblasts (>95% fibroblasts) and the efficiency of *Ets2* conditional knockout (Fig. S1D).

**Figure 3:**
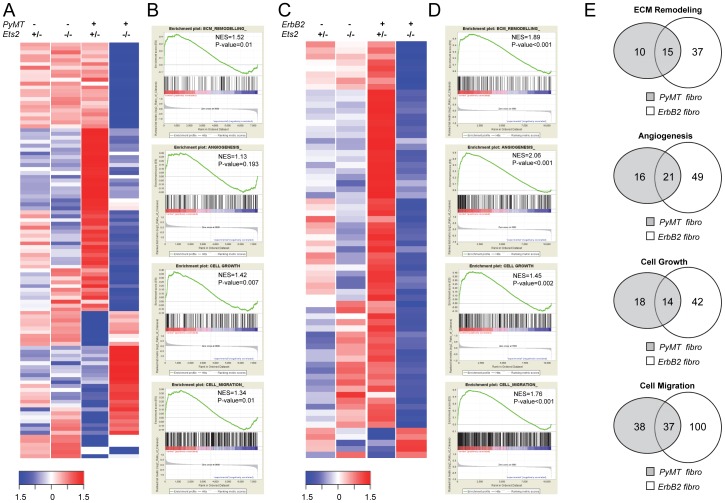
*Ets2* regulates tumor fibroblast specific transcription program. **A.** Heatmap representing expression levels of 107 upregulated and downregulated genes in *PyMT;Ets2^db/loxP^* vs. *PyMT;Fsp-Cre;Ets2^db/loxP^* fibroblasts in all indicated genotypes harvested from 9 week old mice (n = 1, Log fold change>2, Negative Log P value (NLP)>4.5). **B.** GSEA plots depicting ECM remodeling, angiogenesis, cell growth and cell migration to be enriched in *PyMT;Ets2^db/loxP^* fibroblasts as compared to *PyMT;Fsp-Cre;Ets2^db/loxP^* fibroblasts. NES: normalized enrichment score. **C.** Heatmap representing expression levels of 69 upregulated and downregulated genes in *ErbB2;Ets2^db/loxP^* vs. *ErbB2;Fsp-Cre;Ets2^db/loxP^* fibroblasts in all indicated genotypes harvested from 16 week old mice (n = 1, Log fold change>2). **D.** GSEA plots depicting ECM remodeling, angiogenesis, cell growth and cell migration to be enriched in *ErbB2;Ets2^db/loxP^* fibroblasts as compared to *ErbB2;Fsp-Cre;Ets2^db/loxP^* fibroblasts. NES: normalized enrichment score. **E.** Venn diagrams depicting the number of common genes in the leading edge subset of indicated GO categories enriched in *PyMT;Ets2^db/loxP^* fibroblasts (gray circles) and *ErbB2;Ets2^db/loxP^* fibroblasts (white circles).

To identify *Ets2* target genes specifc to tumor fibroblasts, we compared expression changes between normal fibroblasts at both ages (*Fsp-Cre;Ets2^db/loxP^* vs. *Ets2^db/loxP^*) and tumor associated fibroblasts from both models (TAFs; *PyMT;Fsp-Cre;Ets2^db/loxP^* vs. *PyMT;Ets2^db/loxP^* and *ErbB2;Fsp-Cre;Ets2^db/loxP^* vs. *ErbB2;Ets2^db/loxP^* ). Comparison of normal fibroblasts from 9-10 week old mice identified only 22 genes to be differentially expressed upon *Ets2* inactivation (fold change>4 and negative log p-value (NLP)>4.5, Fig. S2A and Table S1). In contrast, when the *PyMT* oncongene was present in epithelial cells, 107 genes were differentially expressed with *Ets2* ablation in fibroblasts (fold change>4 and NLP>4.5, Fig. 3A, Fig. S2A and Table S2). Consistent with a tumor-specific role for *Ets2*, only 4 genes were in common between normal and tumor fibroblasts (Fig. S2A). Our analysis using samples from the *ErbB2* model yielded a similar tumor specific *Ets2-* dependent gene expression pattern, with 15 genes identified as differentially expressed in normal *Ets2* null fibroblasts isolated from 16-week old mice compared to controls, whereas 69 genes changed when *Ets2* was inactivated in *ErbB2* tumor associated fibroblasts (Fig. 3C, Fig. S2C, Table S3 and Table S4). In the *ErbB2* model 1 gene was commonly mis-regulated in between normal and tumor fibroblasts. (Fig. S2C). Quantitative real-time PCR (qRT-PCR) was used to confirm the microarray data, and>85% of the expression changes were verified in at least two independent sets of RNA from tumor fibroblasts isolated from either genetic model (Fig. S2E, S2F).

Gene set enrichment analysis (GSEA) was used to analyze the data [24]. ECM remodeling, cell migration and cell growth-related gene sets were found to be significantly differentially expressed in *PyMT* tumor fibroblasts compared to cells lacking *Ets2* (Fig. 3B). Angiogenesis was another biological process affected by *Ets2* deletion, but the trend did not reach statistical significance (Fig. 3B). GSEA analysis of the *ErbB2* model identified ECM remodeling, cell migration, cell growth-related genes and angiogenesis to be significantly differentially regulated in tumor associated fibroblasts compared to *Ets2* null fibroblasts (Fig. 3D). To determine whether the same genes were driving the enrichment of these biological processes in the two different breast cancer models, the number of overlapping genes present in the leading edge subset for each gene ontology (GO) category was determined (Fig. 3E). Interestingly, between 15–20% of genes present in the leading edge subset of GO categories in the *PyMT* samples were also present in the leading edge in the *ErbB2* samples. This indicates that although *Ets2* may have unique targets that are specific to a particular oncogene, a significant portion of targets are regulated similarly by Ets2 independent of the oncogene. Manual annotation of differentially expressed genes in fibroblasts from both PyMT and ErbB2 models confirmed that genes known to be involved in angiogenesis and ECM remodeling were targets of *Ets2* (Fig. S2B and S2D).

### *Ets2* in Fibroblasts Promotes Angiogenesis in a Cell Autonomous Manner

**Figure 4:**
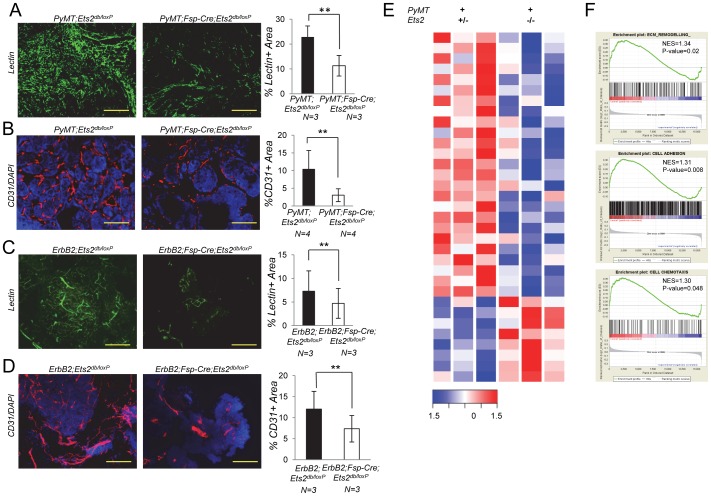
*Ets2* in fibroblasts controls tumor angiogenesis. **A.** Left: *in vivo* tumor vasculature visualized by intracardiac injection of FITC lectin (green). Scale bar, 200 µm. Right: graph represents percent lectin positive area quantified using ImageJ, Fiji(n = 3, bars represent means ± SD, ** P<0.01, Welch’s t-test assuming unequal variance). **B.** Left: immunofluorescence staining for CD31 (red) in mammary gland tumors from 10 week old *PyMT;Ets2^db/loxP^* and *PyMT;Fsp-Cre;Ets2^db/loxP^* mice. Scale bar, 200 µm. Slides were counterstained with DAPI (blue). Right: graph represents percent CD31 positive area quantified using ImageJ, Fiji (n = 3, bars represent means ± SD, **P<0.01, Welch’s t-test assuming unequal variance). **C.** Left: i*n vivo* tumor vasculature visualized by intrafemoral injection of DyLight 594 lectin (green). Scale bar, 200 µm. Right: graph represents percent lectin positive area quantified using ImageJ, Fiji (n = 3, bars represent means ± SD, ** P<0.01, Welch’s t-test assuming unequal variance). **D.** Left: immunofluorescence staining for CD31 (red) in mammary gland tumors from 16 week old *ErbB2;Ets2^db/loxP^* and *ErbB2;Fsp-Cre;Ets2^db/loxP^* mice. Scale bar, 200 µm. Slides were counterstained with DAPI (blue). Right: graph represents percent CD31 positive area quantified using ImageJ, Fiji (n = 3, bars represent means ± SD, **P<0.01, Welch’s t-test assuming unequal variance). **E.** Heatmap depicting the genes significantly differentially regulated in endothelial cells isolated from *PyMT;Ets2^db/loxP^* vs. *PyMT;Fsp-Cre;Ets2^db/loxP^* mammary glands harvested at 9 weeks (n = 3, fold change>2, *P≤0.05). **F.** GSEA plots depicting ECM remodeling, cell adhesion and cell chemotaxis to be enriched in endothelial cells isolated from *PyMT;Ets2^db/loxP^* mammary glands as compared to *PyMT;Fsp-Cre;Ets2^db/loxP^* mammary glands. NES: normalized enrichment score.

To verify whether *Ets2* deletion in fibroblasts compromised angiogenesis as predicted by the microarray results, tumor vasculature was quantified by intracardiac or femoral vein injection of tomato lectin [33]. This analysis revealed a marked decrease in the vascular network in *Ets2*-deleted versus non-deleted control tumors in both the *PyMT* and *ErbB2* models (P<0.01, Fig. 4A, C). Additionally, staining using the endothelial cell specific marker CD31 revealed a decrease in the recruitment of new vasculature in *PyMT* or *ErbB2* tumors lacking *Ets2* in fibroblasts compared to controls (P<0.01, Fig. 4B, D, see S3A and S3B for additional representative images).

To better understand how *Ets2* function in tumor associated fibroblasts promotes angiogenesis, we isolated endothelial cells from the mammary glands of 9-week old *PyMT;Ets2^db/loxP^* and *PyMT;Fsp-Cre;Ets2^db/^* ^loxP^ mice and performed gene expression profiling on isolated endothelial cell RNA (Affymetrix Mouse Genome 430 2.0 GeneChip platform). Our comparison of endothelial cells from *PyMT;Ets2^db/loxP^* and *PyMT;Fsp-Cre;Ets2^db/^* ^loxP^ mammary glands (n = 3 biological replicates) identified 33 differentially regulated genes (fold change>2, P<0.05, Fig. 4E, Table S5). Using GSEA we observed differential regulation of genes involved in ECM remodeling, cell adhesion and cell chemotaxis in tumor endothelial cells when *Ets2* was deleted in fibroblasts (Fig. 4F). Although further analysis is required to determine the exact mechanism by which *Ets2* signaling in fibroblasts affects endothelial cells, our results highlight crosstalk among these different cells in the tumor microenvironment.

**Figure 5:**
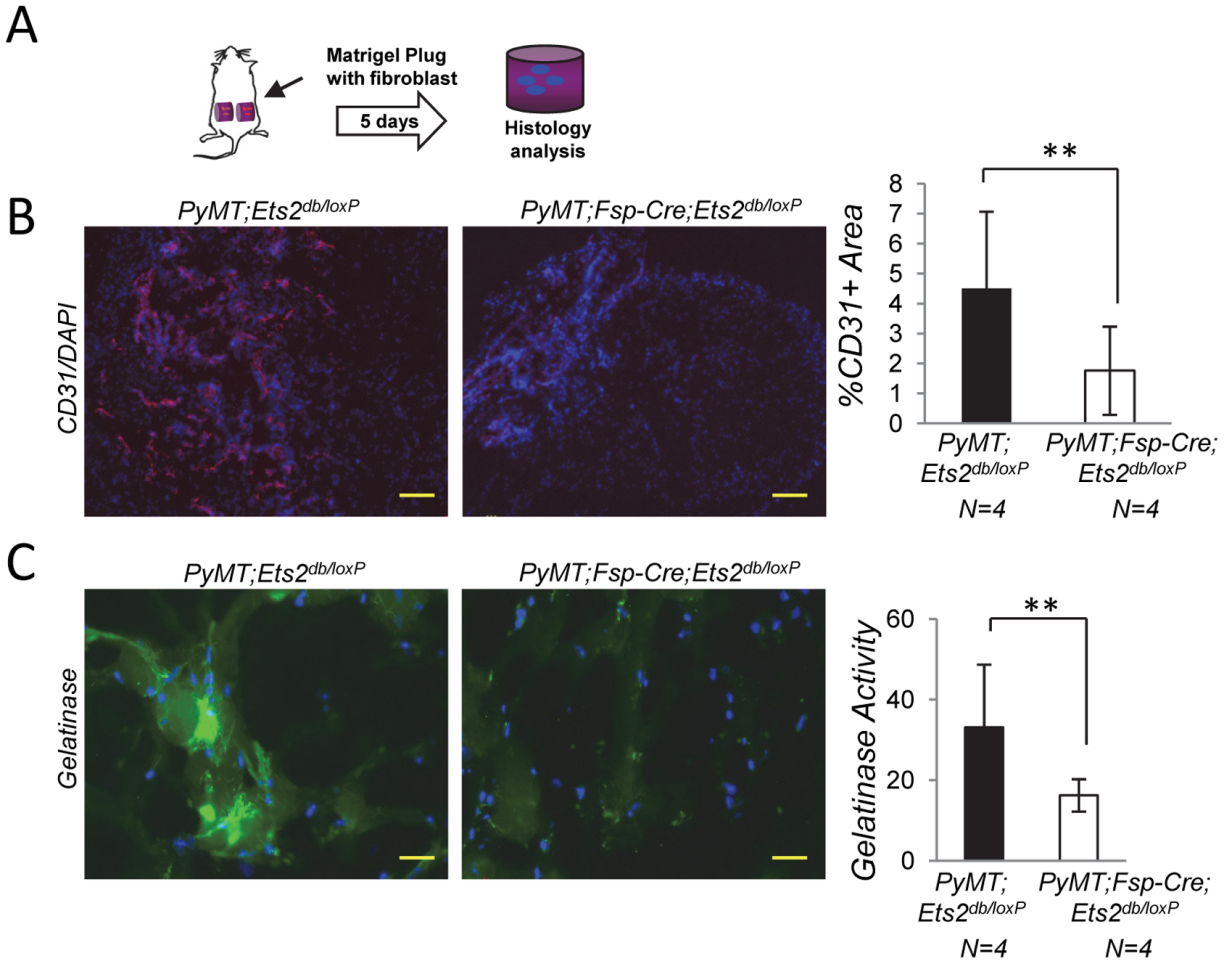
*Ets2* regulation of angiogenesis and ECM remodeling is tumor cell independent. **A.** Experimental strategy of matrigel plug assay. **B.** Left: CD31 staining (red) in matrigel plugs containing either *PyMT;Ets2^db/loxP^* or *PyMT;Fsp-Cre;Ets2^db/loxP^* tumor fibroblasts. Scale bar, 200 µm. Slides were counterstained with DAPI (blue). Right: graph represents percent CD31 positive area quantified using MetaMorph Software (n = 4, bars represent means ± SD, **P<0.01, Welch’s t-test assuming unequal variance). **C.** Right: gelatinase *in situ* zymography for MMP9 activity (green) of matrigel plugs containing primary fibroblasts of indicated genetic groups. Scale bar, 50 µm. Right: graph representing fluorescence intensity quantified using MetaMorph Software (n = 4, bars represent means ± SD, **P<0.01, Welch’s t-test assuming unequal variance).

To experimentally establish whether these effects on angiogenesis were dependent on the cell autonomous function of *Ets2* in fibroblasts, we isolated primary *PyMT* tumor fibroblasts with or without *Ets2* and used these cells for an *in vivo* matrigel plug angiogenesis assay (Fig. 5A) [34], [35]. Matrigel containing *PyMT;Ets2^db/loxP^* and *PyMT;Fsp-Cre;Ets2^db/loxP^* tumor fibroblasts was injected subcutaneously into opposite flanks of syngeneic mice, and plugs were harvested 5 days later for analysis by CD31 staining. These experiments revealed that fibroblasts containing *Ets2* promoted recruitment of endothelial cells more efficiently than fibroblasts lacking *Ets2* (P<0.01, Fig. 5B). The results directly implicate *Ets2* as a driving factor in tumor fibroblasts in *de novo* blood vessel formation. *Mmp9*, a gene directly regulated by *Ets2* [15], is known to play a critical role in angiogenesis, including in the release of active VEGF-A in the ECM [36]. To determine if MMP9 activity was also dependent on *Ets2* function in fibroblasts irrespective of tumor cells, matrigel plugs were analyzed by *in situ* zymography, revealing a 2-fold decrease in MMP9 activity when *Ets2* was deleted in tumor fibroblasts (P<0.01, Fig. 5C). The results of these *in vivo* experiments supports the cell autonomous role of fibroblast *Ets2* in ECM remodeling and angiogenesis.

### *Ets2* Fibroblast Gene Signatures are Represented in Human Breast Cancer Stroma and Correlate with Worsened Patient Outcomes

**Figure 6:**
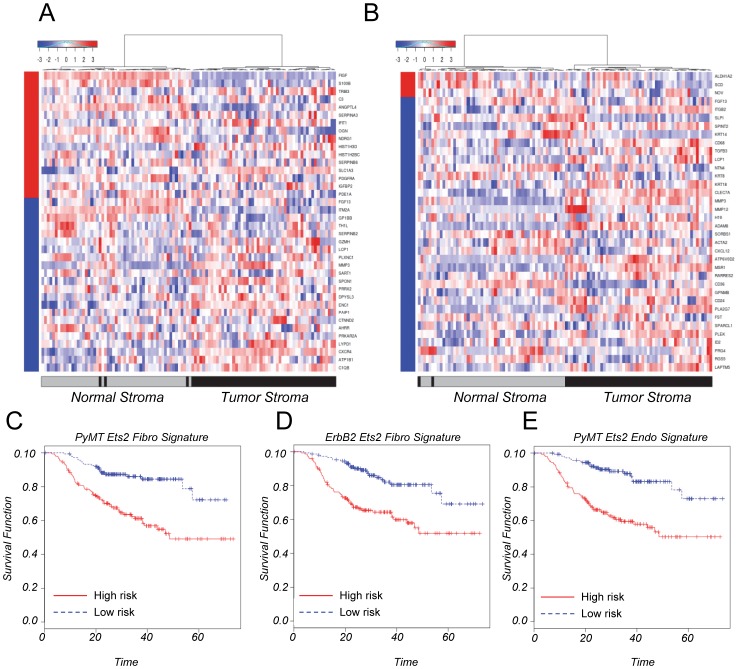
*Ets2* gene signatures are represented in human breast tumor stroma. **A.** Heat map displaying the expression of the human orthologs of the *PyMT* derived 38-gene *Ets2* signature in normal- and tumor-stroma from human breast cancer patients. 16 genes were upregulated (denoted by red bar on the y-axis) and 22 genes were downregulated (denoted by the blue bar on the y-axis) in *Ets2* null tumor fibroblasts. Red and blue regions inside the heat map indicate relative gene expression levels (red, increased and blue, decreased) between the normal and tumor stroma (P = 0.00085, one-sided Wilcoxon rank sum test, based on 10,000 permutations). **B.** Heat map displaying the expression of the human orthologs of the *ErbB2* derived 36-gene *Ets2* signature in normal- and tumor-stroma from human breast cancer patients. 3 genes were upregulated (denoted by red bar on the y-axis) and 33 genes were downregulated (denoted by the blue bar on the y-axis) in *Ets2* null tumor fibroblasts. Red and blue regions inside the heat map indicate relative gene expression levels (red, increased and blue, decreased) between the normal and tumor stroma (P = 0.00004, one-sided Wilcoxon rank sum test, based on 10,000 permutations). **C.** Expression of the 29 *PyMT*-driven *Ets2*-dependent genes present in the NKI whole tumor data set correlates with patient outcome. Kaplan-Meier curves of high risk and low risk groups based on expression of the 29 *Ets2* tumor specific genes (**P<0.0001). **D.** Expression of the 30 *ErbB2*-driven *Ets2*-dependent genes present in the NKI whole tumor data set correlates with patient outcome. Kaplan-Meier curves of high risk and low risk groups based on expression of the 30 *Ets2* tumor specific genes (**P<0.0001). **E.** Expression of the 7 endothelial cell genes dependent on *Ets2* fibroblast signaling present in the NKI whole tumor data set correlates with patient outcome. Kaplan-Meier curves of high risk and low risk groups based on expression of the 7 endothelial cell genes (**P<0.0001).

To evaluate the relevance of our findings to human breast cancer, we compared the *PyMT* driven *Ets2*-dependent fibroblast transcriptome to gene expression data derived from laser-captured human breast tumor stroma and normal stroma [6]. The expression of the human orthologs of 38 genes regulated by *Ets2* in the *PyMT* model (see Materials and Methods) could significantly distinguish human breast tumor stroma from normal stroma compared to 10,000 random gene lists (P = 0.00085, Fig. 6A). Similar results were observed when Principal Component Analysis (PCA) was used (Fig. S4A). Importantly, the *Ets2* gene signature enriched in human tumor stroma correlated with poor patient outcome in two independent and well-annotated whole tumor breast cancer gene expression datasets, the NKI and Stockholm datasets [37], [38], (Fig. 6C and Fig. S4C); the 38-gene *Ets2* expression signature also correlated with patient outcome in the McGill dataset (Fig. S4C).

The *ErbB2* derived *Ets2* tumor fibroblast gene signature was also compared to human breast cancer stroma (see Materials and Methods). The 36 gene signature was found to be significantly enriched in human breast tumor stroma with only 2 patients mis-categorizing (P = 0.00004, Fig. 6B). PCA confirmed the result (Fig. S4B). The expression of these 36 genes was significantly correlated with patient outcomes in the NKI dataset, as well as in the Stockholm and McGill datasets (Fig. 6D and Fig. S4D, respectively).

In contrast to the results we obtained using our fibroblast gene signatures, the endothelial cell gene signature was not able to distinguish human breast tumor stroma from normal stroma (Fig. S5A, S5B). However, 9 variably expressed genes (variance cutoff>0.5, see Materials and Methods) used for the analysis were able to significantly predict patient outcome in the NKI data set, as well as in the Stockholm and McGill datasets (Fig. 6E and Fig. S5C, respectively).

## Conclusions

The tumor fibroblast is critical to breast tumor progression and metastasis, and may provide novel therapeutic opportunities for cancer treatment. In this context, the results presented here provide three significant and inter-related conceptual advances for the breast tumor microenvironment field.

First, the genetic studies demonstrate that *Ets2* regulates an oncogenic gene expression program in tumor stromal fibroblasts that promotes tumor growth. This role of *Ets2* is specific to the stroma since *Ets2* is dispensable for tumor progression in malignant epithelial cells. Although the exact genes regulated by *Ets2* in mammary fibroblasts vary depending on the oncogenic signals coming from the tumor cell, there is a significant overlap of target genes and the overall outcome of increased tumor growth and tumor angiogenesis remain consistent with both oncogenes tested. The activation of *Ets2* stromal fibroblast-specific expression programs represents one mechanism by which the tumor co-opts the microenvironment to serve in its progression to malignancy.

The lack of *Ets2* function in the tumor cells shown here is consistent with a previous report from the Oshima lab [13]. Careful examination of this earlier study indicated that the activity of *MMTV-Cre* utilized was highly mosaic and thus inefficient recombination of the DNA binding region of this *Ets2* allele could account for the apparent lack of *Ets2* function in epithelial cells. In the current work, we used a different *Ets2* conditional allele targeting exons 3–5 configured so that only one floxed allele needs to undergo recombination mediated by *MMTV-Cre*. Our study, ensuring more efficient recombination of the conditional *Ets2* allele, unequivocally demonstrates that *Ets2* is not required in mammary tumor cells in the *PyMT* model employed in both studies. Functional redundancy of *Ets2* with other *Ets*-family members and/or collaborating transcription factors could account for the lack of a significant effect on tumor growth in epithelial cells. Studies in colon cancer mouse models and more recently in human prostate cancer samples provide evidence that *Ets2* can also function as a tumor suppressor gene [39], [40], [41]. In either scenario, cell context is critical in determining whether *Ets2* promotes tumor progression.

A second significant finding presented here is that the consequence of inactivating this *Ets2*-driven stromal program is tumor specific, sparing normal development of the mammary gland. Comparisons with human stromal gene expression demonstrate the mouse *Ets2* pathway functions in human breast cancer progression and correlates with worsened patient outcomes. The results of the matrigel plug assay results imply that fibroblasts acquire a tumor associated “memory” likely dependent on epigenetic changes in these cells, and responsible for the constitutive activation of fibroblasts. Targeting *Ets2*-dependent DNA and histone modifications in tumor associated fibroblasts may lead to more effective treatments in patients with breast tumors without toxicity to normal tissues [42].

Finally, the *Ets2* mechanism involves the activation of a gene expression program that leads to increased angiogenesis. Targeting the tumor vasculature remains an attractive therapeutic target. However, current strategies in which single molecules like VEGF are targeted have demonstrated limited success clinically. Our results indicate that the changes in tumor angiogenesis observed *in vivo* are likely regulated by the cumulative action of many *Ets2* target genes acting in concert. Defining and targeting the pathway hubs controlled by *Ets2* provides an alternative strategy that should be more effective at altering multiple cell-cell and cell-matrix interactions required for tumor angiogenesis.

In conclusion, we have identified *Ets2* as a fibroblast-specific effector of tumor growth through a mechanism that involves increased tumor angiogenesis. The results emphasize the potential utility of identifying tumor-dependent, stromal-specific targets for therapeutic intervention in human breast cancer.

## Supporting Information

Figure S1 **A.** X-gal staining of 10 week old mammary glands from *PyMT;Ets2^db/loxP^*, *PyMT;Fsp-Cre;Ets2^db/loxP^* and *PyMT;MMTV-Cre;Ets2^db/loxP^* animals. Scale bar, 50 µm. **B.** Genotyping of *Ets2* allele in epithelial cells from indicated genetic groups. **C.** Western blot analysis of ETS2 protein levels in fibroblasts isolated from *PyMT;Ets2^db/loxP^* and *PyMT;Fsp-Cre;Ets2^db/loxP^* mice, α-tubulin blotted as loading control. **D**. Immunofluorescence staining of cultured mammary fibroblasts from *PyMT;Ets2^db/loxP^* (left panel) and *PyMT;Fsp-Cre;Ets2^db/loxP^* mice (right panel) with vimentin (green) and anti-phospho-Ets2^T72^ (red) antibodies demonstrates an efficient *Fsp-cre*-mediated deletion of *Ets2* in stromal fibroblasts. Cells were counterstained with 4,6-diamidino-2-phenylindole (DAPI) (blue). Scale bar, 50 µm. **E.** Graph representing percentage of cleaved caspase-3 positive tumor cells in *PyMT;Ets2^db/loxP^* and *PyMT;Fsp-Cre;Ets2^db/loxP^* mammary glands (n = 2, bars represent means ± SD, P>0.05, Welch’s t-test assuming unequal variance). **F.** Whole mount carmine staining of inguinal mammary glands from 6 week old *Ets2^db/loxP^* and *Fsp-Cre;Ets2^db/loxP^* mice. L.N., lymph node. Scale bar, 2mm. **G.** Whole mount carmine staining of inguinal mammary glands from 14 days pregnant *Ets2^db/loxP^* and *Fsp-Cre;Ets2^db/loxP^* mice. L.N., lymph node. Scale bar, 2mm. **H.** Whole mount carmine staining of inguinal mammary glands from 6 week old *PyMT;Ets2^db/loxP^* and *PyMT;Fsp-Cre;Ets2^db/loxP^* mice. L.N., lymph node. Scale bar, 2mm.
(TIF)

Figure S2 **A.** Venn diagram depicting the number of similarly regulated genes in normal fibroblasts (*Ets2^db/loxP^* vs. *Fsp-Cre;Ets2^db/loxP^*, gray circle) and tumor fibroblasts (*PyMT;Ets2^db/loxP^* vs. *PyMT;Fsp-Cre;Ets2^db/loxP^*, white circle) harvested from 9 week old mice. **B.** Functional annotation of tumor-specific (*PyMT;Ets2^db/loxP^* vs. *PyMT;Fsp-Cre;Ets2^db/loxP^*) target genes of *Ets2*. **C.** Venn diagram depicting number of similarly regulated genes in normal fibroblasts (*Ets2^db/loxP^* vs. *Fsp-Cre;Ets2^db/loxP^*, gray circle) and tumor fibroblasts (*ErbB2;Ets2^db/loxP^* vs. *ErbB2;Fsp-Cre;Ets2^db/loxP^*, white circle) harvested from 16 week old mice. **D.** Functional annotation of tumor-specific (*ErbB2;Ets2^db/loxP^* vs. *ErbB2;Fsp-Cre;Ets2^db/loxP^*) target genes of *Ets2*. **E, F.** Quantitative RT-PCR analysis of indicated genes in independent primary fibroblasts of indicated genotypes. Gene expression is normalized to *Rpl4* expression, and graphed as fold difference between genotypes (values are means between duplicates of one representative sample ± SD).
(TIF)

Figure S3 **A.** Immunofluorescence staining for CD31 (red) in mammary gland tumors from 10 week old *PyMT;Ets2^db/loxP^* (panels on the left) and *PyMT;Fsp-Cre;Ets2^db/loxP^* mice (panels on the right) shows a decrease in tumor angiogenesis when *Ets2* is deleted in stromal fibroblasts. Scale bar, 200 µm. Slides were counterstained with DAPI (blue). **B.** Immunofluorescence staining for CD31 (red) in mammary gland tumors from 16 week old *ErbB2;Ets2^db/loxP^* (panels on the left) and *ErbB2;Fsp-Cre;Ets2^db/loxP^* mice (panels on the right) shows a decrease in tumor angiogenesis when *Ets2* is deleted in stromal fibroblasts. Scale bar, 200 µm. Slides were counterstained with DAPI (blue).
(TIF)

Figure S4 **A.** Principal Component Analysis (PCA) to show separation of tumor stroma (n = 49, black circles) and normal stroma (n = 52, gray circles) using 38-gene subset *PyMT*-driven *Ets2* signature. **B.** Principal Component Analysis (PCA) to show separation of tumor stroma (n = 49, black circles) and normal stroma (n = 52, gray circles) using 36-gene subset *ErbB2*-driven *Ets2* signature. **C.** Expression of the 38 and 36 *PyMT*-driven *Ets2*-dependent genes present in the McGill (left panel) and Stockholm (right panel) stroma and whole tumor data sets, respectively, correlate with patient outcome. Kaplan-Meier curves of high risk and low risk groups based on expression of the 38 and 36 *Ets2* tumor specific genes (*P<0.05 for McGill data set and *P≤0.05 for Stockholm data set). **D.** Expression of the 36 and 33 *ErbB2*-driven *Ets2*-dependent genes present in the McGill (left panel) and Stockholm (right panel) stroma and whole tumor data sets, respectively, correlate with patient outcome. Kaplan-Meier curves of high risk and low risk groups based on expression of the 36 and 33 *Ets2* tumor specific genes (*P<0.05 for McGill data set and *P≤0.05 for Stockholm data set).
(TIF)

Figure S5 **A.** Heat map displaying the expression of the human orthologs of the *PyMT* derived fibroblast *Ets2*-dependent endothelial cell 9 gene signature in normal- and tumor-stroma from human breast cancer patients. Red and blue regions inside the heat map indicate relative gene expression levels (red, increased and blue, decreased) between the normal and tumor stroma. (P = 0.1014, one-sided Wilcoxon rank sum test, based on 10,000 permutations). **B.** Principal Component Analysis (PCA) to show separation of tumor stroma (n = 49, black circles) and normal stroma (n = 52, gray circles) with 9 gene fibroblast *Ets2* dependent endothelial cell signature. **C.** Expression of 7 *PyMT*-driven *Ets2*-dependent genes in endothelial cells present in the McGill (left panel) and Stockholm (right panel) stroma and whole tumor data sets, respectively, correlate with patient outcome. Kaplan-Meier curves of high risk and low risk groups based on expression of 7 *Ets2* tumor-specific genes in endothelial cells (*P<0.05 for McGill data set and *P<0.05 for Stockholm data set).
(TIF)

Table S1 **Comparison of gene expression changes in fibroblasts from 9–10 week old** ***Ets2^db/loxP^*** **and** ***Fsp-Cre;Ets2^db/loxP^*** **mice identifies 22 genes differentially expressed upon loss of** ***Ets2*** **(Log fold change>2 and negative log p-value (NLP)>4.5).**
(DOCX)

Table S2 **Gene expression analysis of tumor associated fibroblasts from 9–10 week old** ***PyMT;Ets2^db/loxP^*** **and** ***PyMT;Fsp-Cre;Ets2^db/loxP^*** **mice reveals the differential expression of 107 genes with** ***Ets2*** **ablation in fibroblasts (Log fold change>2 and NLP>4.5).**
(DOCX)

Table S3 ** Comparison of gene expression changes between normal fibroblasts from 16 week old** ***Ets2^db/loxP^*** **and** ***Fsp-Cre;Ets2^db/loxP^*** **mice identifies 15 genes regulated by** ***Ets2*** **(Log fold change>2).**
(DOCX)

Table S4 **Gene expression analysis of tumor associated fibroblasts from 16 week old** ***ErbB2;Ets2^db/loxP^*** **and** ***ErbB2;Fsp-Cre;Ets2^db/loxP^*** **mice reveals the differential expression of 69 genes when** ***Ets2*** **is deleted in fibroblasts (Log fold change>2).**
(DOCX)

Table S5 **List of 33 differentially expressed genes in endothelial cells from 9–10 week** ***PyMT;Ets2^db/loxP^*** **and** ***PyMT;Fsp-Cre;Ets2^db/loxP^*** **mice as a consequence of** ***Ets2*** **function in PyMT tumor associated fibroblasts (Log fold change>2).**
(DOCX)

Table S6 **List of qRT-PCR primers and probes used for confirmation of gene expression changes.**
(DOCX)
