# Supplementary figures and images for: Ets2 in Tumor Fibroblasts Promotes Angiogenesis in Breast Cancer
Source: PLoS One. 2013 Aug 16;8(8):e71533. doi: 10.1371/journal.pone.0071533 (PMC3745457; doi:10.1371/journal.pone.0071533)

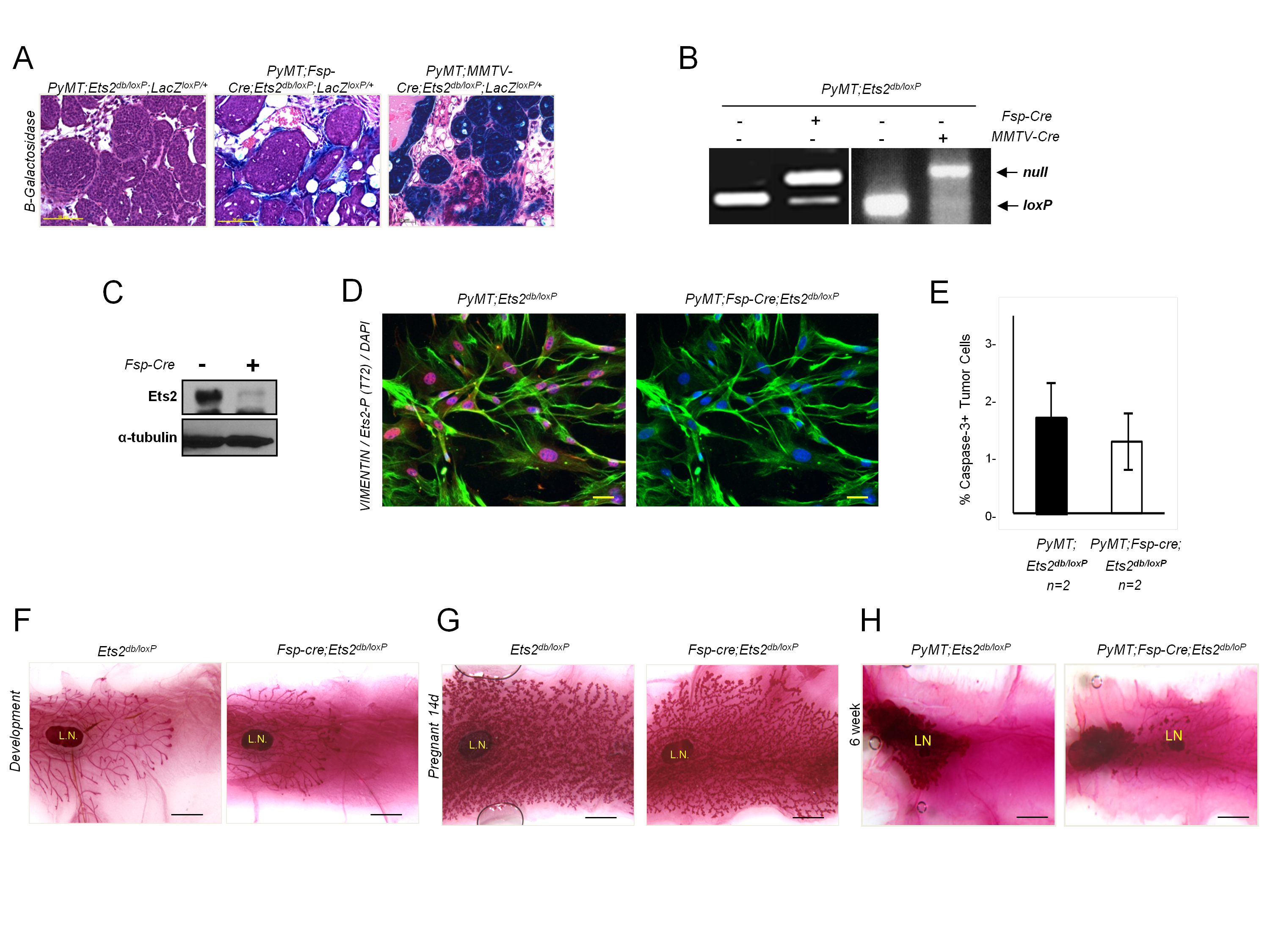

Supplement: Figure S1 — A. X-gal staining of 10 week old mammary glands from PyMT;Ets2db/loxP, PyMT;Fsp-Cre;Ets2db/loxP and PyMT;MMTV-Cre;Ets2db/loxP animals. Scale bar, 50 µm. B. Genotyping of Ets2 allele in epithelial cells from indicated genetic groups. C. Western blot analysis of ETS2 protein levels in fibroblasts isolated from PyMT;Ets2db/loxP and PyMT;Fsp-Cre;Ets2db/loxP mice, α-tubulin blotted as loading control. D. Immunofluorescence staining of cultured mammary fibroblasts from PyMT;Ets2db/loxP (left panel) and PyMT;Fsp-Cre;Ets2db/loxP mice (right panel) with vimentin (green) and anti-phospho-Ets2T72 (red) antibodies demonstrates an efficient Fsp-cre-mediated deletion of Ets2 in stromal fibroblasts. Cells were counterstained with 4,6-diamidino-2-phenylindole (DAPI) (blue). Scale bar, 50 µm. E. Graph representing percentage of cleaved caspase-3 positive tumor cells in PyMT;Ets2db/loxP and PyMT;Fsp-Cre;Ets2db/loxP mammary glands (n = 2, bars represent means ± SD, P>0.05, Welch’s t-test assuming unequal variance). F. Whole mount carmine staining of inguinal mammary glands from 6 week old Ets2db/loxP and Fsp-Cre;Ets2db/loxP mice. L.N., lymph node. Scale bar, 2mm. G. Whole mount carmine staining of inguinal mammary glands from 14 days pregnant Ets2db/loxP and Fsp-Cre;Ets2db/loxP mice. L.N., lymph node. Scale bar, 2mm. H. Whole mount carmine staining of inguinal mammary glands from 6 week old PyMT;Ets2db/loxP and PyMT;Fsp-Cre;Ets2db/loxP mice. L.N., lymph node. Scale bar, 2mm. (TIF) [file pone.0071533.s001.tif]

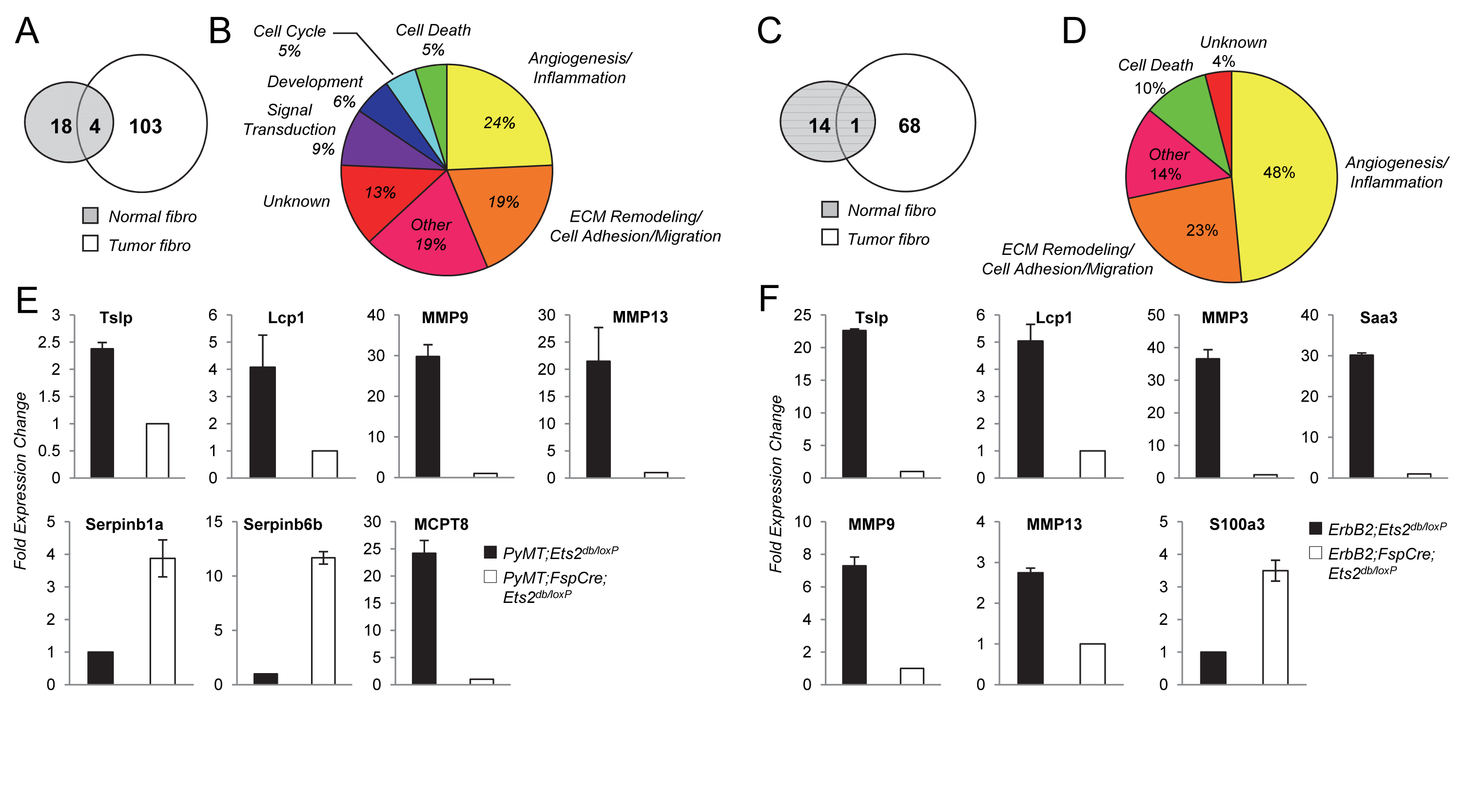

Supplement: Figure S2 — A. Venn diagram depicting the number of similarly regulated genes in normal fibroblasts (Ets2db/loxP vs. Fsp-Cre;Ets2db/loxP, gray circle) and tumor fibroblasts (PyMT;Ets2db/loxP vs. PyMT;Fsp-Cre;Ets2db/loxP, white circle) harvested from 9 week old mice. B. Functional annotation of tumor-specific (PyMT;Ets2db/loxP vs. PyMT;Fsp-Cre;Ets2db/loxP) target genes of Ets2. C. Venn diagram depicting number of similarly regulated genes in normal fibroblasts (Ets2db/loxP vs. Fsp-Cre;Ets2db/loxP, gray circle) and tumor fibroblasts (ErbB2;Ets2db/loxP vs. ErbB2;Fsp-Cre;Ets2db/loxP, white circle) harvested from 16 week old mice. D. Functional annotation of tumor-specific (ErbB2;Ets2db/loxP vs. ErbB2;Fsp-Cre;Ets2db/loxP) target genes of Ets2. E, F. Quantitative RT-PCR analysis of indicated genes in independent primary fibroblasts of indicated genotypes. Gene expression is normalized to Rpl4 expression, and graphed as fold difference between genotypes (values are means between duplicates of one representative sample ± SD). (TIF) [file pone.0071533.s002.tif]

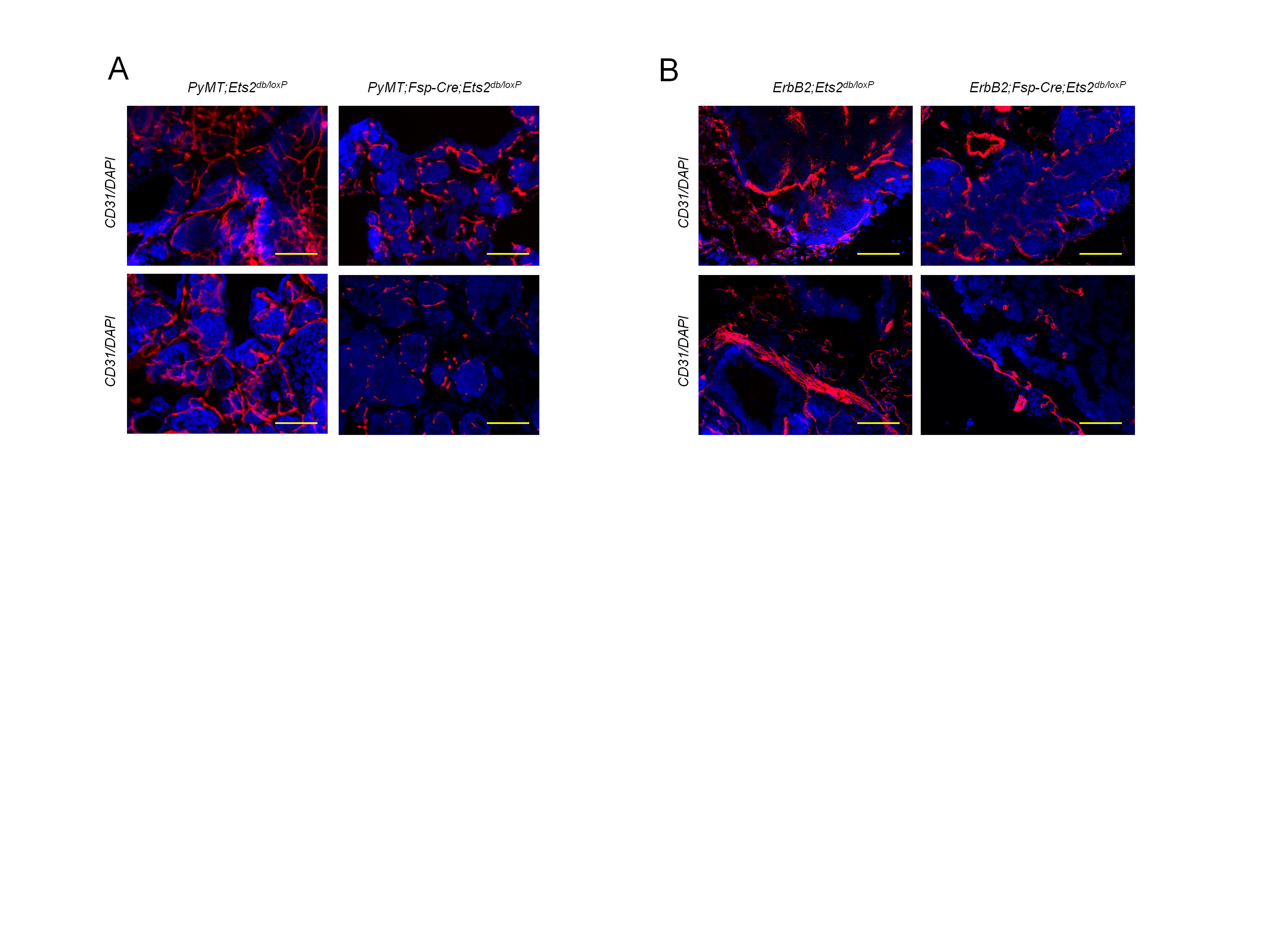

Supplement: Figure S3 — A. Immunofluorescence staining for CD31 (red) in mammary gland tumors from 10 week old PyMT;Ets2db/loxP (panels on the left) and PyMT;Fsp-Cre;Ets2db/loxP mice (panels on the right) shows a decrease in tumor angiogenesis when Ets2 is deleted in stromal fibroblasts. Scale bar, 200 µm. Slides were counterstained with DAPI (blue). B. Immunofluorescence staining for CD31 (red) in mammary gland tumors from 16 week old ErbB2;Ets2db/loxP (panels on the left) and ErbB2;Fsp-Cre;Ets2db/loxP mice (panels on the right) shows a decrease in tumor angiogenesis when Ets2 is deleted in stromal fibroblasts. Scale bar, 200 µm. Slides were counterstained with DAPI (blue). (TIF) [file pone.0071533.s003.tif]

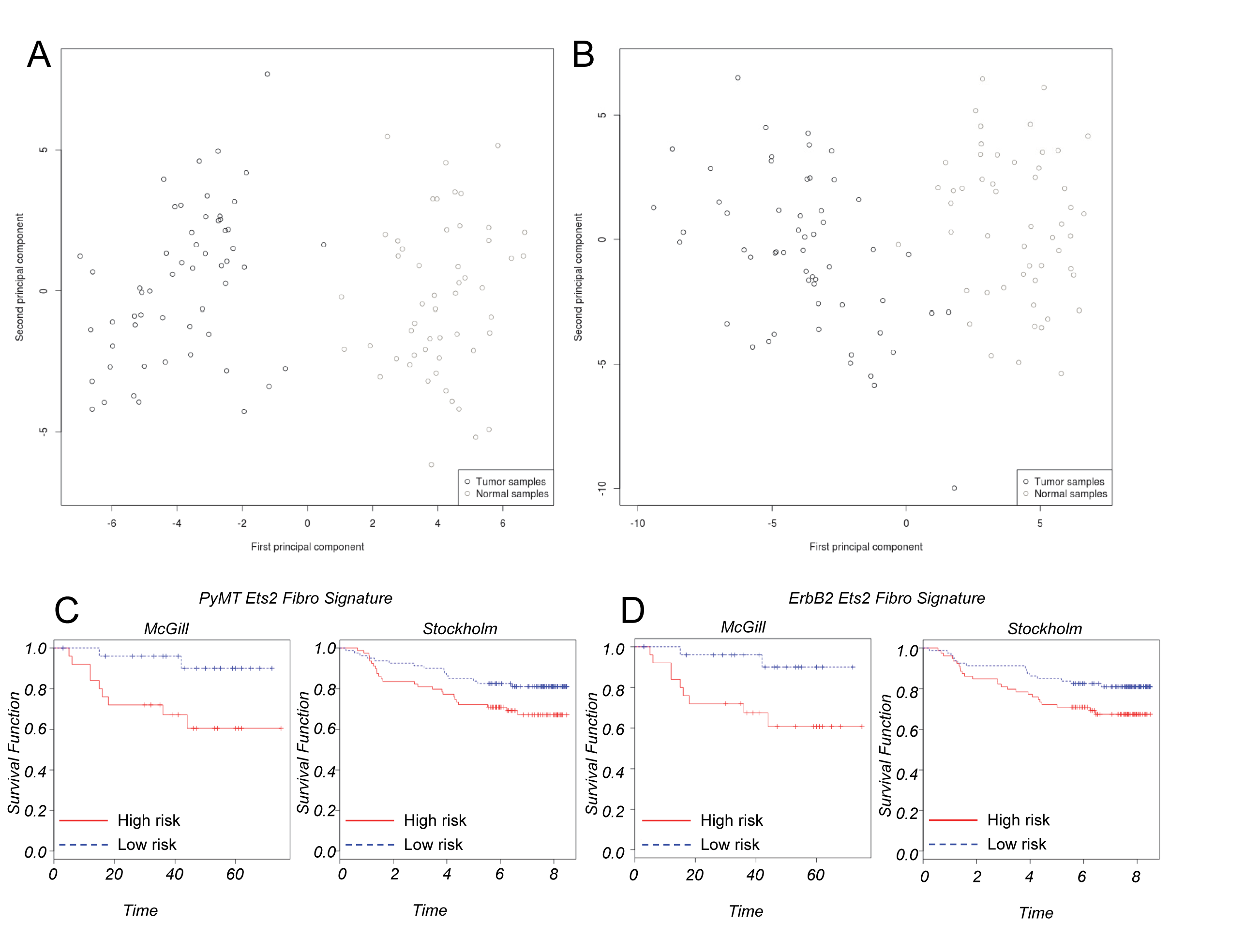

Supplement: Figure S4 — A. Principal Component Analysis (PCA) to show separation of tumor stroma (n = 49, black circles) and normal stroma (n = 52, gray circles) using 38-gene subset PyMT-driven Ets2 signature. B. Principal Component Analysis (PCA) to show separation of tumor stroma (n = 49, black circles) and normal stroma (n = 52, gray circles) using 36-gene subset ErbB2-driven Ets2 signature. C. Expression of the 38 and 36 PyMT-driven Ets2-dependent genes present in the McGill (left panel) and Stockholm (right panel) stroma and whole tumor data sets, respectively, correlate with patient outcome. Kaplan-Meier curves of high risk and low risk groups based on expression of the 38 and 36 Ets2 tumor specific genes (*P<0.05 for McGill data set and *P≤0.05 for Stockholm data set). D. Expression of the 36 and 33 ErbB2-driven Ets2-dependent genes present in the McGill (left panel) and Stockholm (right panel) stroma and whole tumor data sets, respectively, correlate with patient outcome. Kaplan-Meier curves of high risk and low risk groups based on expression of the 36 and 33 Ets2 tumor specific genes (*P<0.05 for McGill data set and *P≤0.05 for Stockholm data set). (TIF) [file pone.0071533.s004.tif]

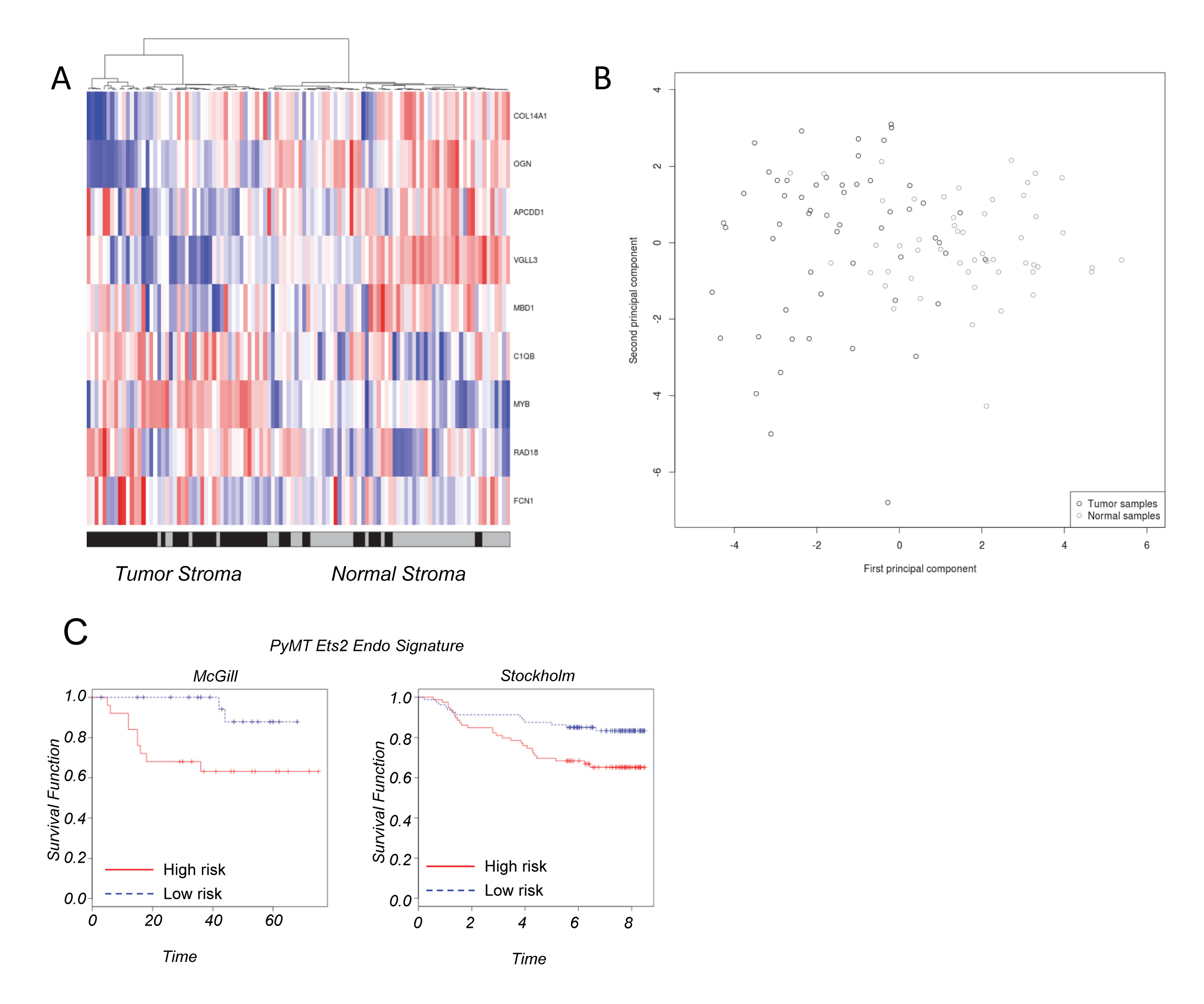

Supplement: Figure S5 — A. Heat map displaying the expression of the human orthologs of the PyMT derived fibroblast Ets2-dependent endothelial cell 9 gene signature in normal- and tumor-stroma from human breast cancer patients. Red and blue regions inside the heat map indicate relative gene expression levels (red, increased and blue, decreased) between the normal and tumor stroma. (P = 0.1014, one-sided Wilcoxon rank sum test, based on 10,000 permutations). B. Principal Component Analysis (PCA) to show separation of tumor stroma (n = 49, black circles) and normal stroma (n = 52, gray circles) with 9 gene fibroblast Ets2 dependent endothelial cell signature. C. Expression of 7 PyMT-driven Ets2-dependent genes in endothelial cells present in the McGill (left panel) and Stockholm (right panel) stroma and whole tumor data sets, respectively, correlate with patient outcome. Kaplan-Meier curves of high risk and low risk groups based on expression of 7 Ets2 tumor-specific genes in endothelial cells (*P<0.05 for McGill data set and *P<0.05 for Stockholm data set). (TIF) [file pone.0071533.s005.tif]
