# Supplementary material for: Ets2 in Tumor Fibroblasts Promotes Angiogenesis in Breast Cancer
Source: PLoS One. 2013 Aug 16;8(8):e71533. doi: 10.1371/journal.pone.0071533 (PMC3745457; doi:10.1371/journal.pone.0071533)
Supplement: Table S1 — Comparison of gene expression changes in fibroblasts from 9–10 week old Ets2db/loxP and Fsp-Cre;Ets2db/loxP mice identifies 22 genes differentially expressed upon loss of Ets2 (Log fold change>2 and negative log p-value (NLP)>4.5). (DOCX) [file pone.0071533.s006.docx]

**Table S1. 22 genes Regulated by Ets2 in normal 9-10-week mammary fibroblasts.**

| **Probeset** | **NLP** | **Ets2+-N** | **Ets2--N** | **GENE** | **Log Fold Change** |
| --- | --- | --- | --- | --- | --- |
| 1449832_at | 5.44 | 3.316 | 5.407 | 1700091H14Rik | 2.091 |
| 1416200_at | 7.13 | 7.332 | 9.767 | 9230117N10Rik | 2.435 |
| 1427974_s_at | 4.60 | 4.051 | 6.844 | Cacna1d | 2.793 |
| 1417795_at | 4.60 | 3.982 | 6.909 | Chl1 | 2.927 |
| 1455435_s_at | 4.60 | 6.056 | 8.090 | Chdh | 2.034 |
| 1437458_x_at | 6.28 | 5.818 | 8.395 | Clu | 2.577 |
| 1422592_at | 5.44 | 6.199 | 3.742 | Ctnnd2 | -2.457 |
| 1450839_at | 4.60 | 6.286 | 8.338 | D0H4S114 | 2.052 |
| 1419332_at | 7.13 | 6.062 | 8.797 | Egfl6 | 2.735 |
| 1424007_at | 8.82 | 9.857 | 12.086 | Gdf10 | 2.229 |
| 1421973_at | 4.60 | 4.157 | 6.282 | Gfra1 | 2.125 |
| 1423171_at | 7.97 | 6.338 | 8.866 | Gpr88 | 2.528 |
| 1427300_at | 7.13 | 9.024 | 11.511 | Lhx8 | 2.487 |
| 1423607_at | 8.82 | 9.261 | 11.622 | Lum | 2.361 |
| 1424234_s_at | 4.60 | 4.603 | 2.554 | Meox2 | -2.049 |
| 1425521_at | 7.13 | 7.711 | 4.244 | Paip1 | -3.467 |
| 1418759_at | 4.60 | 3.798 | 6.354 | Ptpn20 | 2.556 |
| 1421856_at | 4.60 | 3.141 | 7.397 | S100a3 | 4.256 |
| 1450826_a_at | 7.97 | 7.676 | 9.698 | Saa3 | 2.022 |
| 1448301_s_at | 8.82 | 5.549 | 7.763 | Serpinb1a | 2.214 |
| 1417979_at | 7.13 | 8.460 | 4.252 | Tnmd | -4.208 |
| 1456319_at | 4.60 | 4.621 | 7.518 | X83313 | 2.897 |

NLP: Negative log P value. Expression level is represented in log2. Fold change is log2.
