# Supplementary material for: Ets2 in Tumor Fibroblasts Promotes Angiogenesis in Breast Cancer
Source: PLoS One. 2013 Aug 16;8(8):e71533. doi: 10.1371/journal.pone.0071533 (PMC3745457; doi:10.1371/journal.pone.0071533)
Supplement: Table S2 — Gene expression analysis of tumor associated fibroblasts from 9–10 week old PyMT;Ets2db/loxP and PyMT;Fsp-Cre;Ets2db/loxP mice reveals the differential expression of 107 genes with Ets2 ablation in fibroblasts (Log fold change>2 and NLP>4.5). (DOCX) [file pone.0071533.s007.docx]

**Table S2. 107 genes regulated by Ets2 in 9-10 week PyMT tumor associated fibroblasts.**

| **Probeset** | **NLP** | **Ets+-T** | **Ets--T** | **GENE** | **Log Fold Change** |
| --- | --- | --- | --- | --- | --- |
| 1449902_at | 4.60 | 5.058 | 2.917 | 1110058A15Rik | -2.141 |
| 1451382_at | 4.60 | 5.682 | 8.101 | 1810008K03Rik | 2.419 |
| 1420548_a_at | 4.60 | 6.133 | 3.395 | 2310008H09Rik | -2.738 |
| 1427202_at | 4.60 | 5.505 | 7.511 | 4833442J19Rik | 2.006 |
| 1428333_at | 4.60 | 6.598 | 4.554 | 6530401D17Rik | -2.044 |
| 1418979_at | 8.82 | 3.064 | 6.651 | 9030611N15Rik | 3.587 |
| 1420796_at | 4.60 | 5.771 | 2.841 | Ahrr | -2.930 |
| 1422415_at | 4.60 | 7.304 | 3.238 | Ang2 | -4.066 |
| 1417130_s_at | 4.60 | 5.883 | 8.054 | Angptl4 | 2.171 |
| 1417732_at | 6.28 | 3.706 | 5.758 | Anxa8 | 2.052 |
| 1419435_at | 7.13 | 5.261 | 7.464 | Aox1 | 2.203 |
| 1439036_a_at | 4.60 | 5.891 | 3.777 | Atp1b1 | -2.114 |
| 1454838_s_at | 6.28 | 6.788 | 4.533 | AW548124 | -2.255 |
| 1424652_at | 4.60 | 6.352 | 3.659 | BC014699 | -2.693 |
| 1434366_x_at | 4.60 | 6.656 | 4.615 | C1qb | -2.041 |
| 1423954_at | 8.82 | 4.778 | 7.268 | C3 | 2.490 |
| 1424713_at | 5.54 | 4.607 | 6.686 | Calml4 | 2.079 |
| 1418126_at | 4.60 | 5.784 | 3.763 | Ccl5 | -2.021 |
| 1448698_at | 5.44 | 7.677 | 10.097 | Ccnd1 | 2.420 |
| 1415868_at | 4.60 | 6.814 | 4.237 | Cct4 | -2.577 |
| 1419703_at | 6.28 | 7.129 | 4.512 | Col5a3 | -2.617 |
| 1455269_a_at | 4.60 | 5.576 | 3.430 | Coro1a | -2.146 |
| 1422592_at | 5.44 | 6.986 | 4.553 | Ctnnd2 | -2.433 |
| 1448710_at | 6.28 | 9.845 | 5.430 | Cxcr4 | -4.415 |
| 1422812_at | 4.60 | 6.180 | 3.430 | Cxcr6 | -2.750 |
| 1418507_s_at | 7.97 | 9.074 | 7.046 | D130043N08Rik | -2.028 |
| 1424625_a_at | 5.44 | 4.821 | 2.392 | Dennd1a | -2.429 |
| 1438789_s_at | 4.60 | 6.654 | 4.121 | Dpysl3 | -2.533 |
| 1425295_at | 4.60 | 7.552 | 5.432 | Ear11 | -2.120 |
| 1424306_at | 4.60 | 6.931 | 4.293 | Elovl4 | -2.638 |
| 1420964_at | 4.60 | 6.900 | 4.799 | Enc1 | -2.101 |
| 1419131_at | 4.60 | 3.298 | 5.426 | F13b | 2.128 |
| 1450779_at | 4.60 | 6.179 | 3.607 | Fabp7 | -2.572 |
| 1418497_at | 4.60 | 5.389 | 3.049 | Fgf13 | -2.340 |
| 1438953_at | 4.60 | 8.631 | 10.706 | Figf | 2.075 |
| 1422977_at | 5.44 | 5.528 | 3.442 | Gp1bb | -2.086 |
| 1420344_x_at | 7.97 | 9.081 | 4.644 | Gzmd | -4.437 |
| 1450171_x_at | 8.82 | 10.486 | 7.261 | Gzme | -3.225 |
| 1449455_at | 4.60 | 8.378 | 5.619 | Hck | -2.759 |
| 1425398_at | 4.60 | 5.209 | 8.510 | Hist1h2bc | 3.301 |
| 1453573_at | 4.60 | 5.653 | 7.839 | Hist1h3d | 2.186 |
| 1425874_at | 8.82 | 8.900 | 6.600 | Hoxc13 | -2.300 |
| 1450783_at | 4.60 | 5.228 | 7.337 | Ifit1 | 2.109 |
| 1449025_at | 4.60 | 4.047 | 6.787 | Ifit3 | 2.740 |
| 1454159_a_at | 7.13 | 6.031 | 8.923 | Igfbp2 | 2.892 |
| 1450091_at | 4.60 | 3.321 | 5.340 | Ighmbp2 | 2.019 |
| 1423608_at | 4.60 | 7.297 | 4.169 | Itm2a | -3.128 |
| 1418156_at | 7.97 | 8.548 | 10.775 | Kcne4 | 2.227 |
| 1450185_a_at | 4.60 | 4.495 | 6.574 | Kcnj15 | 2.079 |
| 1427679_at | 7.13 | 6.760 | 4.582 | Lats1 | -2.178 |
| 1415983_at | 4.60 | 8.037 | 4.878 | Lcp1 | -3.159 |
| 1437477_at | 5.44 | 5.222 | 3.201 | Lrrfip1 | -2.021 |
| 1431569_a_at | 4.60 | 8.368 | 4.929 | Lypd1 | -3.439 |
| 1449965_at | 7.13 | 8.637 | 4.490 | Mcpt8 | -4.147 |
| 1424481_s_at | 4.60 | 6.608 | 3.397 | MGC38735 | -3.211 |
| 1418377_a_at | 4.60 | 6.619 | 4.369 | MGI:1353606 | -2.250 |
| 1448416_at | 8.82 | 11.993 | 9.060 | Mgp | -2.933 |
| 1420450_at | 7.13 | 8.947 | 5.556 | Mmp10 | -3.391 |
| 1417256_at | 4.60 | 9.058 | 4.732 | Mmp13 | -4.326 |
| 1418945_at | 8.82 | 9.540 | 6.874 | Mmp3 | -2.666 |
| 1416298_at | 4.60 | 7.744 | 3.603 | Mmp9 | -4.141 |
| 1422557_s_at | 8.82 | 10.284 | 8.215 | Mt1 | -2.069 |
| 1417155_at | 7.97 | 6.549 | 4.090 | Mycn | -2.459 |
| 1419391_at | 4.60 | 2.841 | 5.329 | Myog | 2.488 |
| 1450976_at | 4.60 | 3.152 | 6.114 | Ndrg1 | 2.962 |
| 1419663_at | 4.60 | 8.021 | 10.364 | Ogn | 2.343 |
| 1425521_at | 7.13 | 7.862 | 4.253 | Paip1 | -3.609 |
| 1449298_a_at | 5.44 | 3.596 | 7.049 | Pde1a | 3.453 |
| 1421916_at | 6.28 | 4.272 | 6.834 | Pdgfra | 2.562 |
| 1427038_at | 5.44 | 7.026 | 2.797 | Penk1 | -4.229 |
| 1421403_at | 4.60 | 6.331 | 3.969 | Pi15 | -2.362 |
| 1427327_at | 8.82 | 8.634 | 6.461 | Pilra | -2.173 |
| 1419280_at | 5.44 | 5.583 | 3.504 | Pip5k2a | -2.079 |
| 1448961_at | 6.28 | 4.363 | 6.642 | Plscr2 | 2.279 |
| 1450905_at | 4.60 | 7.728 | 3.379 | Plxnc1 | -4.349 |
| 1428494_a_at | 4.60 | 7.306 | 5.255 | Polr2i | -2.051 |
| 1433691_at | 6.28 | 4.050 | 7.110 | Ppp1r3c | 3.060 |
| 1427414_at | 4.60 | 5.441 | 3.256 | Prkar2a | -2.185 |
| 1432331_a_at | 5.44 | 6.984 | 4.668 | Prrx2 | -2.316 |
| 1448899_s_at | 4.60 | 6.739 | 4.342 | Rad51ap1 | -2.397 |
| 1450107_a_at | 5.44 | 8.156 | 5.803 | Renbp | -2.353 |
| 1424143_a_at | 6.28 | 7.948 | 5.869 | Ris2 | -2.079 |
| 1455893_at | 7.97 | 3.941 | 8.618 | Rspo2 | 4.677 |
| 1421856_at | 4.60 | 3.188 | 7.896 | S100a3 | 4.708 |
| 1434342_at | 4.60 | 2.869 | 5.277 | S100b | 2.408 |
| 1450826_a_at | 7.97 | 8.169 | 5.679 | Saa3 | -2.490 |
| 1423010_at | 6.28 | 6.630 | 3.735 | Sacs | -2.895 |
| 1448658_at | 4.60 | 6.223 | 4.163 | Sart1 | -2.060 |
| 1427020_at | 7.97 | 6.793 | 4.367 | Scara3 | -2.426 |
| 1419100_at | 8.82 | 5.676 | 7.891 | Serpina3n | 2.215 |
| 1416318_at | 8.82 | 5.119 | 8.263 | Serpinb1a | 3.144 |
| 1419082_at | 4.60 | 8.875 | 3.864 | Serpinb2 | -5.011 |
| 1422804_at | 8.82 | 6.615 | 9.836 | Serpinb6b | 3.221 |
| 1452031_at | 7.97 | 5.150 | 7.208 | Slc1a3 | 2.058 |
| 1422648_at | 5.44 | 5.092 | 7.120 | Slc7a2 | 2.028 |
| 1418425_at | 4.60 | 6.937 | 3.559 | Sp7 | -3.378 |
| 1424415_s_at | 6.28 | 7.940 | 4.261 | Spon1 | -3.679 |
| 1456355_s_at | 5.44 | 6.700 | 4.154 | Srr1 | -2.546 |
| 1417725_a_at | 4.60 | 6.977 | 3.521 | Sssca1 | -3.456 |
| 1420894_at | 4.60 | 6.629 | 3.756 | Tgfbr1 | -2.873 |
| 1416198_at | 4.60 | 5.493 | 3.036 | Th1l | -2.457 |
| 1417109_at | 4.60 | 5.704 | 8.109 | Tinagl | 2.405 |
| 1450731_s_at | 5.44 | 10.485 | 8.320 | Tnfrsf21 | -2.165 |
| 1456225_x_at | 4.60 | 4.904 | 7.419 | Trib3 | 2.515 |
| 1450004_at | 4.60 | 8.700 | 3.607 | Tslp | -5.093 |
| 1434243_s_at | 4.60 | 7.533 | 5.053 | Tomm70a | -2.480 |
| 1437281_x_at | 4.60 | 6.330 | 4.138 | Xab2 | -2.192 |

NLP: Negative log P value. Expression level is represented in log2. Fold change is log2.
