# Supplementary material for: Ets2 in Tumor Fibroblasts Promotes Angiogenesis in Breast Cancer
Source: PLoS One. 2013 Aug 16;8(8):e71533. doi: 10.1371/journal.pone.0071533 (PMC3745457; doi:10.1371/journal.pone.0071533)
Supplement: Table S3 — Comparison of gene expression changes between normal fibroblasts from 16 week old Ets2db/loxP and Fsp-Cre;Ets2db/loxP mice identifies 15 genes regulated by Ets2 (Log fold change>2). (DOCX) [file pone.0071533.s008.docx]

**Table S3. 15 genes Regulated by Ets2 in wild-type 16-week mammary fibroblasts.**

| **Probeset** | **Ets2+-N** | **Ets2--N** | **GENE** | **Log Fold Change** |
| --- | --- | --- | --- | --- |
| 1452244_at | 8.316 | 6.095 | 6330406I15 | -2.222 |
| 1441054_at | 4.194 | 7.528 | Apol8 | 3.334 |
| 1439036_a_at | 5.264 | 7.391 | Atp1b1 | 2.127 |
| 1448182_a_at | 5.614 | 8.058 | Cd24a | 2.443 |
| 1437689_x_at | 6.477 | 8.763 | Clu /// LO | 2.285 |
| 1416579_a_at | 5.381 | 7.833 | Epcam | 2.452 |
| 1423935_x_at | 5.543 | 8.268 | Krt14 | 2.725 |
| 1448169_at | 5.212 | 8.781 | Krt18 | 3.569 |
| 1423691_x_at | 5.920 | 9.397 | Krt8 | 3.477 |
| 1457040_at | 7.896 | 5.877 | Lgi2 | -2.019 |
| 1423413_at | 7.337 | 5.257 | Ndrg1 | -2.080 |
| 1426851_a_at | 8.195 | 5.727 | Nov | -2.469 |
| 1427760_s_at | 8.296 | 12.398 | Prl2c2 /// | 4.102 |
| 1421856_at | 6.380 | 9.834 | S100a3 | 3.454 |
| 1448201_at | 8.378 | 6.328 | Sfrp2 | -2.050 |

Expression level is represented in log2. Fold change is log2.
