# Supplementary material for: Ets2 in Tumor Fibroblasts Promotes Angiogenesis in Breast Cancer
Source: PLoS One. 2013 Aug 16;8(8):e71533. doi: 10.1371/journal.pone.0071533 (PMC3745457; doi:10.1371/journal.pone.0071533)
Supplement: Table S4 — Gene expression analysis of tumor associated fibroblasts from 16 week old ErbB2;Ets2db/loxP and ErbB2;Fsp-Cre;Ets2db/loxP mice reveals the differential expression of 69 genes when Ets2 is deleted in fibroblasts (Log fold change>2). (DOCX) [file pone.0071533.s009.docx]

**Table S4. 69 genes regulated by Ets2 in 16 week ErbB2 tumor associated fibroblasts.**

| **Probeset** | **Ets2+-T** | **Ets2--T** | **GENE** | **Log Fold Change** |
| --- | --- | --- | --- | --- |
| 1444105_at | 8.891 | 6.703 | Acta2 | -2.189 |
| 1415927_at | 8.790 | 5.997 | Actc1 /// | -2.793 |
| 1416871_at | 10.108 | 7.837 | Adam8 | -2.271 |
| 1422789_at | 4.634 | 6.788 | Aldh1a2 | 2.154 |
| 1444176_at | 7.523 | 5.201 | Atp6v0d2 | -2.322 |
| 1451620_at | 7.855 | 5.720 | C1ql3 | -2.135 |
| 1442082_at | 8.509 | 5.898 | C3ar1 | -2.610 |
| 1420249_s_at | 9.297 | 6.454 | Ccl6 | -2.842 |
| 1448182_a_at | 7.610 | 4.816 | Cd24a | -2.794 |
| 1423166_at | 10.441 | 6.692 | Cd36 | -3.750 |
| 1449164_at | 10.560 | 8.214 | Cd68 | -2.347 |
| 1420804_s_at | 9.257 | 6.773 | Clec4d | -2.484 |
| 1425951_a_at | 7.235 | 4.437 | Clec4n | -2.798 |
| 1420699_at | 8.831 | 6.367 | Clec7a | -2.464 |
| 1437689_x_at | 9.460 | 5.013 | Clu /// LO | -4.447 |
| 1455660_at | 7.119 | 4.826 | Csf2rb | -2.293 |
| 1448591_at | 11.493 | 8.995 | Ctss | -2.498 |
| 1448823_at | 10.494 | 7.687 | Cxcl12 | -2.807 |
| 1420512_at | 4.902 | 7.231 | Dkk2 | 2.329 |
| 1416579_a_at | 8.480 | 5.625 | Epcam | -2.855 |
| 1450779_at | 8.197 | 6.153 | Fabp7 | -2.044 |
| 1460555_at | 7.195 | 5.178 | Fam65b | -2.017 |
| 1418497_at | 8.686 | 6.184 | Fgf13 | -2.502 |
| 1434458_at | 9.834 | 7.743 | Fst | -2.092 |
| 1436530_at | 11.177 | 8.881 | Gm11428 | -2.296 |
| 1420394_s_at | 10.982 | 8.870 | Gp49a /// | -2.112 |
| 1448303_at | 10.950 | 8.913 | Gpnmb | -2.037 |
| 1448194_a_at | 8.133 | 5.684 | H19 | -2.449 |
| 1418645_at | 8.792 | 6.641 | Hal | -2.151 |
| 1435176_a_at | 8.326 | 5.898 | Id2 | -2.427 |
| 1450678_at | 9.305 | 7.163 | Itgb2 | -2.142 |
| 1423935_x_at | 8.260 | 4.927 | Krt14 | -3.332 |
| 1448169_at | 8.523 | 5.007 | Krt18 | -3.517 |
| 1423952_a_at | 8.517 | 6.283 | Krt7 | -2.234 |
| 1423691_x_at | 8.884 | 5.158 | Krt8 | -3.726 |
| 1436905_x_at | 9.285 | 7.243 | Laptm5 | -2.042 |
| 1415983_at | 9.005 | 6.963 | Lcp1 | -2.042 |
| 1449153_at | 11.614 | 7.869 | Mmp12 | -3.745 |
| 1417256_at | 8.436 | 5.788 | Mmp13 | -2.647 |
| 1418945_at | 12.262 | 8.292 | Mmp3 | -3.970 |
| 1419598_at | 7.320 | 5.186 | Ms4a6d | -2.133 |
| 1448061_at | 8.978 | 6.546 | Msr1 | -2.432 |
| 1452651_a_at | 8.231 | 5.428 | Myl1 | -2.803 |
| 1448371_at | 8.335 | 5.304 | Mylpf | -3.031 |
| 1419391_at | 7.313 | 5.182 | Myog | -2.130 |
| 1426852_x_at | 7.299 | 10.244 | Nov | 2.945 |
| 1450791_at | 8.516 | 5.961 | Nppb | -2.555 |
| 1439794_at | 7.937 | 5.436 | Ntn4 | -2.501 |
| 1448995_at | 8.939 | 6.268 | Pf4 | -2.671 |
| 1430700_a_at | 8.600 | 5.880 | Pla2g7 | -2.720 |
| 1448749_at | 8.240 | 6.151 | Plek | -2.089 |
| 1449824_at | 7.805 | 4.871 | Prg4 | -2.934 |
| 1427760_s_at | 9.371 | 6.409 | Prl2c2 /// | -2.962 |
| 1428538_s_at | 11.150 | 8.619 | Rarres2 | -2.532 |
| 1415905_at | 7.656 | 5.605 | Reg1 | -2.051 |
| 1417466_at | 7.845 | 5.289 | Rgs5 | -2.556 |
| 1421856_at | 5.205 | 9.678 | S100a3 | 4.473 |
| 1450826_a_at | 10.230 | 7.995 | Saa3 | -2.235 |
| 1415823_at | 9.518 | 11.636 | Scd2 | 2.118 |
| 1448377_at | 11.186 | 8.613 | Slpi | -2.572 |
| 1440311_at | 7.293 | 4.839 | Sorbs1 | -2.454 |
| 1416114_at | 7.940 | 5.684 | Sparcl1 | -2.256 |
| 1438968_x_at | 7.750 | 5.362 | Spint2 | -2.388 |
| 1417455_at | 11.957 | 9.842 | Tgfb3 | -2.115 |
| 1424967_x_at | 6.752 | 4.175 | Tnnt2 | -2.577 |
| 1426175_a_at | 8.335 | 6.275 | Tpsab1 | -2.060 |
| 1450004_at | 9.868 | 4.220 | Tslp | -5.647 |
| 1450792_at | 10.267 | 8.043 | Tyrobp | -2.225 |
| 1419417_at | 8.700 | 6.632 | Vegfc | -2.068 |

Expression level is represented in log2. Fold change is log2.
