# Supplementary material for: Ets2 in Tumor Fibroblasts Promotes Angiogenesis in Breast Cancer
Source: PLoS One. 2013 Aug 16;8(8):e71533. doi: 10.1371/journal.pone.0071533 (PMC3745457; doi:10.1371/journal.pone.0071533)
Supplement: Table S5 — List of 33 differentially expressed genes in endothelial cells from 9–10 week PyMT;Ets2db/loxP and PyMT;Fsp-Cre;Ets2db/loxP mice as a consequence of Ets2 function in PyMT tumor associated fibroblasts (Log fold change>2). (DOCX) [file pone.0071533.s010.docx]

**Table S5. 33 genes differentially expressed in endothelial cells from 9-10 week *PyMT; ets2^db/loxP^* vs. *PyMT; Fsp-cre; ets2^db/loxP^* as a consequence of Ets2 function in PyMT tumor associated fibroblasts.**

| **Probeset** | **P-value** | **Average Ets2+-T** | **Average Ets2--T** | **Gene_Symbol** | **Log Fold Change** |
| --- | --- | --- | --- | --- | --- |
| 1442358_at | 0.02 | 6.83 | 5.76 | AA409587 | -1.0662 |
| 1443639_at | 0.04 | 4.64 | 5.84 | Apcdd1 | 1.206 |
| 1435761_at | 0.03 | 7.57 | 6.45 | BC100530 | -1.116 |
| 1427434_at | 0.03 | 6.64 | 5.62 | Birc1f | -1.0252 |
| 1437726_x_at | 0.02 | 12.04 | 10.75 | C1qb | -1.2953 |
| 1420249_s_at | 0.00 | 9.63 | 8.48 | Ccl6 | -1.154 |
| 1419684_at | 0.02 | 10.45 | 9.28 | Ccl8 | -1.1642 |
| 1427168_a_at | 0.01 | 7.76 | 6.60 | Col14a1 | -1.1584 |
| 1449218_at | 0.02 | 8.33 | 7.21 | Cox8b | -1.1168 |
| 1435578_s_at | 0.00 | 3.71 | 5.48 | Dab1 | 1.7726 |
| 1435940_at | 0.05 | 6.44 | 5.39 | Dclk1 | -1.0483 |
| 1459725_s_at | 0.02 | 10.25 | 8.70 | Dcpp1 | -1.5478 |
| 1435943_at | 0.02 | 7.18 | 6.05 | Dpep1 | -1.1327 |
| 1450779_at | 0.02 | 4.51 | 5.64 | Fabp7 | 1.1377 |
| 1418243_at | 0.01 | 7.77 | 6.52 | Fcna | -1.2505 |
| 1456601_x_at | 0.00 | 7.79 | 6.51 | Fxyd2 | -1.2723 |
| 1439793_at | 0.01 | 6.14 | 4.99 | Gja3 | -1.1414 |
| 1422224_at | 0.01 | 4.89 | 5.92 | Gm9880 | 1.0315 |
| 1425763_x_at | 0.05 | 10.35 | 8.52 | Igh | -1.8272 |
| 1439239_at | 0.03 | 6.93 | 5.72 | Lin7b | -1.2124 |
| 1453678_at | 0.02 | 6.54 | 5.35 | Mbd1 | -1.1932 |
| 1460462_at | 0.00 | 6.05 | 7.71 | Med18 | 1.6565 |
| 1450194_a_at | 0.00 | 6.80 | 5.80 | Myb | -1.0057 |
| 1436188_a_at | 0.00 | 6.49 | 5.24 | Ndrg4 | -1.2457 |
| 1419663_at | 0.03 | 7.51 | 6.48 | Ogn | -1.021 |
| 1428751_at | 0.04 | 4.50 | 5.51 | Pacrg | 1.0099 |
| 1420798_s_at | 0.01 | 6.10 | 4.51 | Pcdha1 | -1.5971 |
| 1443954_at | 0.01 | 6.37 | 7.58 | Rad18 | 1.2077 |
| 1456822_at | 0.02 | 6.31 | 5.28 | Rad23b | -1.0315 |
| 1438306_at | 0.02 | 5.45 | 4.32 | Rnf180 | -1.1357 |
| 1458813_at | 0.01 | 7.15 | 5.83 | Scn5a | -1.3204 |
| 1457275_at | 0.02 | 7.28 | 8.46 | Synm | 1.1782 |
| 1455299_at | 0.00 | 6.49 | 4.72 | Vgll3 | -1.7784 |

Expression level is represented in log2. Fold change is log2.
