# Supplementary material for: Ets2 in Tumor Fibroblasts Promotes Angiogenesis in Breast Cancer
Source: PLoS One. 2013 Aug 16;8(8):e71533. doi: 10.1371/journal.pone.0071533 (PMC3745457; doi:10.1371/journal.pone.0071533)
Supplement: Table S6 — List of qRT-PCR primers and probes used for confirmation of gene expression changes. (DOCX) [file pone.0071533.s011.docx]

**Table S6**: **qRT-PCR Primers and Probe List**

| **Gene ID** | **Left primer** | **Right primer** | **PROBE #** |
| --- | --- | --- | --- |
| LCP1 | tcaacagacgggctgattc | agagcagccttgtcaagca | #4 |
| MCPT8 | tgttcctgctcctggtcct | tggggtttggactctgtacc | #83 |
| MMP3 | ttgttctttgatgcagtcagc | gatttgcgccaaaagtgc | #7 |
| MMP9 | acgacatagacggcatcca | gctgtggttcagttgtggtg | #19 |
| MMP13 | cagtctccgaggagaaactatga | ggactttgtcaaaaagagctcag | #62 |
| S100a3 | gggacacccagttggtagg | gcacacgatggcagctact | #1 |
| Saa3 | atgctcgggggaactatgat | acagcctctctggcatcg | #26 |
| Serpinb1a | tgctggtgaatgccatctact | tcttcactgtttttgtgtctttcc | #52 |
| Serpinb6b | ggtccttgtgaatgccatct | tgcacaggtttcaccacatc | #7 |
| Tslp | catgacctgactggagatttga | atgttttgtcggggagtgaa | #71 |
